# Transcriptional Regulation of the Human MGP Promoter: Identification of Downstream Repressors

**DOI:** 10.3390/ijms252312597

**Published:** 2024-11-23

**Authors:** Helena Caiado, M. Leonor Cancela, Natércia Conceição

**Affiliations:** 1 Center of Marine Sciences (CCMAR), University of Algarve, 8005-139 Faro, Portugal; helena.isabel.caiado@gmail.com (H.C.); lcancela@ualg.pt (M.L.C.); 2 Faculty of Medicine and Biomedical Sciences, University of Algarve, 8005-139 Faro, Portugal; 3 Algarve Biomedical Center, University of Algarve, 8005-139 Faro, Portugal

**Keywords:** MGP, transcriptional regulation, methylation, YY1, GATA1, C/EBPα

## Abstract

Matrix Gla protein (MGP) is a vitamin K-dependent γ-carboxylated protein that was initially identified as a physiological inhibitor of ectopic calcification, primarily affecting cartilage and the vascular system. Mutations in the *MGP* gene were found to be responsible for the Keutel syndrome, a condition characterized by abnormal calcifications in the cartilage, lungs, brain, and vascular system. *MGP* has been shown to be dysregulated in several tumors, including cervical, ovarian, urogenital, and breast cancers. Using bioinformatic approaches, transcription factor binding sites (TFBSs) containing CpG dinucleotides were identified in the *MGP* promoter, including those for YY1, GATA1, and C/EBPα. We carried out functional tests using transient transfections with a luciferase reporter assay, primarily for the transcription factors YY1, GATA1, C/EBPα, and RUNX2. By co-transfection analysis, we found that YY1, GATA1, and C/EBPα repressed the *MGP* promoter. Furthermore, the co-transfection with RUNX2 activated the *MGP* promoter. In addition, *MGP* expression is negatively or positively correlated with the studied TFs’ expression levels in several cancer types. This study provides novel insights into *MGP* regulation by demonstrating that YY1, GATA1, and C/EBPα are negative regulators of the *MGP* promoter, and DNA methylation may influence their activity. The dysregulation of these mechanisms in cancer should be further elucidated.

## 1. Introduction

Matrix Gla protein (MGP) is a member of the family of vitamin K-dependent Gla proteins mainly associated with the extracellular matrix. The *MGP* gene located on chromosome 12p12.3 [1] is highly expressed by vascular smooth muscle cells (VSMCs) [2] and chondrocytes [3]. The MGP function was clarified through the identification of mutations in *MGP* that lead to the Keutel syndrome [4]. Recently, two new heterozygous variants were identified in the *MGP* gene altering the Cys19 residue in the translated protein responsible for autosomal dominant spondyloepiphyseal dysplasia [5]. Consistent with the functional importance of MGP in physiology, deregulated expression of *MGP* has been reported to correlate with the development of various other pathologies including colorectal, ovarian, lung, and breast cancers [6].

Despite the knowledge of the involvement of MGP in diverse cellular events, the mechanisms regulating the expression of the *MGP* gene remain largely unknown. The regulation of the *MGP* promoter has been investigated in previous studies and some putative transcription factor binding sites (TFBSs) were reported, such as retinoic acid receptor (RAR) [7], vitamin D receptor (VDR), cyclic adenosine monophosphate receptor (cAMP receptor) [8], and parathyroid hormone (PTH) [9,10], but only a few were shown to be functional: activating protein-1 (AP1) [11], fibroblast growth factor 2 (FGF2) [12], homeobox C8 (HOXC8) [13], and early growth response-1 (Egr-1) [14].

It is known that cytosine methylation, in CpG sites, might change the spatial structure of DNA including at TFBSs, and therefore may affect transcriptional regulation by changes in the affinity of TFs binding to DNA [15].

Since the precise mechanisms involved in *MGP* regulation are unknown, the main objective of this work was to obtain novel knowledge regarding *MGP* transcriptional and epigenetic regulation and to understand the molecular mechanisms involved. For that, we characterized the putative TFBSs in the *MGP* promoter overlapping CpG sites and analyzed their functionality. To better understand the functional consequences of TF binding, we analyzed the results of transient transfection promoter activity assays carried out in the HEK293 and MCF-7 cell lines. In each assay, we compared the activity of the *MGP* wild-type promoter construct with that of a mutant promoter construct in which the predicted TF binding site was abolished. The mutations were chosen to mutate only the CG nucleotides or to abolish TF binding by mutating as many as six nucleotides in the most informative positions, that is, those making the greatest contribution to the TF-DNA binding free energy. Our results indicate that the transcription factors Yin Yang 1 (YY1), GATA binding protein 1 (GATA1), CCAAT/enhancer binding protein alpha (C/EBPα), and Runt-related transcription factor 2 (RUNX2) contribute to the regulation of the *MGP* gene at the transcriptional level.

## 2. Results

### 2.1. In Silico Analysis of Transcription Binding Sites and CpG Sites Identified in the MGP Promoter

We started by identifying possible CpG islands or CpG sites located in the human *MGP* promoter, through the analysis of the USCS genome browser. This public database, available online, allows us to visualize CpG ID probes for specific CpG sites within a given gene, generated by the Illumina Infinium Human Methylation 450 Bead Array platform.

**Figure 1:**
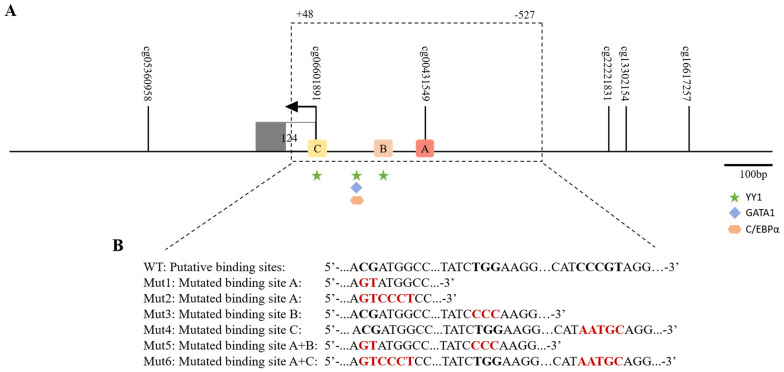
Putative transcription factor binding sites in human *MGP* promoter region. (**A**) Regulatory elements in the promoter region were searched using several bioinformatic tools. Geometric shapes represent the transcription factor binding sites for YY1 (star), GATA1 (diamond), and C/EBPα (hexagon). The smaller, closed arrow represents the transcription start site. Each small, vertical line represents a single CpG site. Exons and promoter regions are in scale. (**B**) Summary of the various mutations introduced in the identified binding sites. The different mutants produced are shown below in the wild-type (WT) sequence for the three putative binding sites. Mutated nucleotides are shown in red. In construct Mut1, for site A, YY1, GATA1, and C/EBPα sites were mutated in the nucleotides CG. In construct Mut2, for site A, in YY1, GATA1, and C/EBPα sites, additional nucleotides were mutated. In construct mut3, the binding for YY1 in site B was altered as indicated. In construct mut4, for site C, the YY1 site was mutated as shown. In construct mut5, both sites A and B were mutated as shown, and in construct mut6, both sites A and C were mutated as shown.

The results indicated the presence of six CpG sites located in or around the *MGP* gene, with four located in the promoter region, one in the transcription start site according to the sequence from NCBI (Reference Sequence: NG_023331.1), and one located in the first intron (Figure 1). Since DNA methylation is one of the factors that may influence gene expression and interfere with the binding of transcription factors, the promoter region of the *MGP* gene (MGP560) spanning from −527 to +48 relative to the transcriptional start site, containing the CpG sites cg00431549 and cg06601891 (Figure 1A), was used to search for possible transcription factor binding sites (TFBSs) overlapping the CpG, through an in silico approach using three different online prediction databases.

Since *MGP* is dysregulated in several types of cancers, in our analysis, we selected transcription factors known from the literature to be involved in carcinogenesis processes. The results showed the presence of three putative binding sites for YY1, GATA1, and C/EBPα overlapping the CpG site cg00431549 and one binding site for YY1 overlapping the CpG site cg06601891 (Figure 1A).

### 2.2. Functional Analysis of MGP Promoter Activity Due to the Predicted Transcription Factors

To assess the promoter activity of the *MGP* gene due to the selected transcription factors, we generated a luciferase reporter construct containing the *MGP* promoter (MGP560), as well as constructs containing site-directed mutations for the respective putative binding sites for YY1, GATA1, and C/EBPα (Figure 1B). These fragments were then co-transfected with YY1, GATA1, and C/EBPα expression plasmids and their respective controls.

#### 2.2.1. Transcription Factor YY1 Represses *MGP* Luciferase Promoter Activity

**Figure 2:**
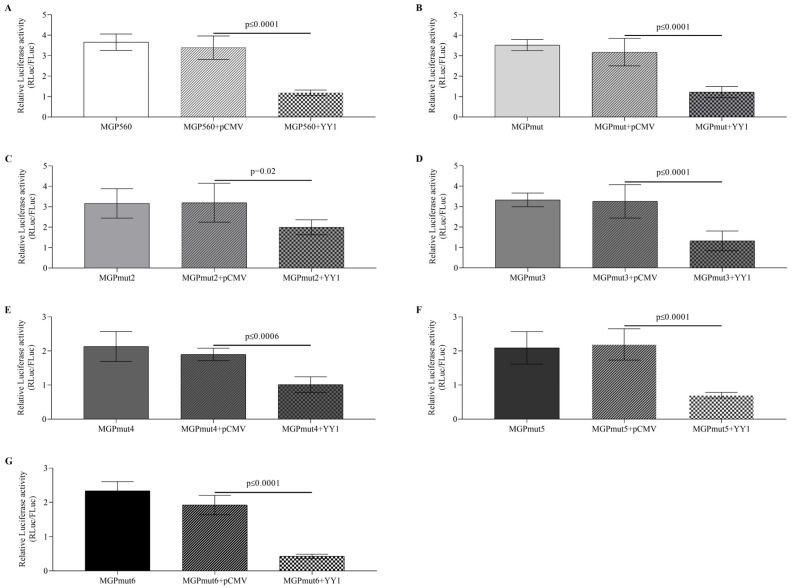
Effect of the YY1 transcription factor in the *MGP* promoter activity. The *MGP* promoter fragment (MGP560) (**A**), the construct MGP560mut1 (**B**), the construct MGP560Mut2 (**C**), the construct MGP560Mut3 (**D**), the construct MGP560Mut4 (**E**), the construct MGP560Mut5 (**F**), and the construct MGP560Mut6 (**G**) were shown to be repressed in the presence of the transcription factor YY1. Data are presented as mean ± SD of at least five independent experiments. Statistical significance was determined by a one-way ANOVA statistical test with Tukey correction; *p*-values were considered significant when *p* ≤ 0.05.

Co-transfection of the *MGP* promoter fragment (MGP560) with the YY1 expressing vector resulted in a significant 2.9-fold reduction in the luciferase activity (Figure 2A) over the control (the MGP560 promoter fragment co-transfected with the pCMV empty vector), suggesting a possible regulation by this transcription factor. To determine whether the CpG site is important for the binding of the transcription factor YY1, we performed a site-directed mutation targeting the CG nucleotides containing the putative binding site for this transcription factor. However, when we co-transfected the expressing vector containing YY1 with the MGP560mut1 construct, we observed similar repression of 2.6-fold in the promoter activity, suggesting that the mutation of the CpG site was not sufficient to restore the luciferase promoter activity (Figure 2B).

Since the repression was visible after mutating the CG (MGP560mut1), we performed a new site-directed mutation of six nucleotides targeting the entire core sequence of YY1 (MGP560mut2). The results showed repression of 1.7-fold over the control with this mutation (Figure 2C), suggesting that the repressive effect on the *MGP* promoter by YY1 could be due to the binding of this transcription factor in another binding site(s) within this promoter fragment.

To explore the hypothesis of another putative binding site(s) for the YY1 transcription factor in the *MGP* promoter region under analysis, we searched through an in silico approach for new possible binding sites for YY1. The results showed another putative binding site for YY1, located at the position −144 to −141 in addition to the one located in the transcription start site (Figure 1). First, we explored the effect of this YY1 binding site in the *MGP* promoter luciferase activity by mutating it (MGP560mut3). However, a similar decrease of 2.5-fold over the control in the luciferase activity of the construct fragment MGP560mut3 was observed upon co-transfection with the expression vector carrying YY1 (Figure 2D).

Next, we evaluated if the effect of YY1 in the *MGP* luciferase activity was due to binding at the transcription start site (site C in Figure 1A) by mutating it (MGP560mut4). The results showed again a decrease in the luciferase activity of 1.9-fold over the control, suggesting that this site might not be a binding site for the YY1 transcription factor (Figure 2E).

To investigate the potential negative synergistic effect of YY1 binding at the identified binding sites, we generated two constructs: MGP560mut5 (with mutations in both sites A and B) and MGP560mut6 (with mutations in both sites A and C) (Figure 1B). The results show a repressive effect on *MGP* promoter activity in both constructs when co-transfected with the YY1 expressing vector (Figure 2F,G), suggesting that YY1 does not bind to any of these sites. While the exact binding site could not be identified, these findings still support a role for YY1 in regulating basal *MGP* transcription.

#### 2.2.2. Transcription Factor GATA1 Binds to the *MGP* Promoter Repressing Its Activity

**Figure 3:**
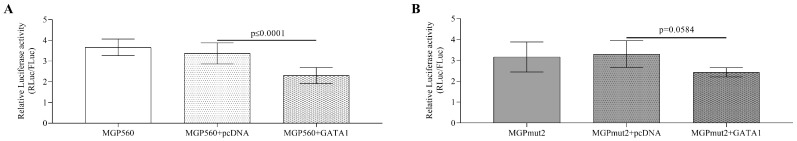
Effect of the GATA1 transcription factor in *MGP* promoter activity. The *MGP* promoter fragment MGP560 (**A**) and the construct MGP560Mut2 (**B**) were co-transfected with the transcription factor GATA1. Data are presented as mean ± SD of at least five independent experiments. Statistical significance was determined by a one-way ANOVA statistical test with Tukey’s correction; *p*-values were considered significant when *p* ≤ 0.05.

Luciferase activity was significantly decreased when the GATA1 expression vector was co-transfected with *MGP* promoter fragment MGP560, suggesting that GATA1 might be a negative regulator of *MGP* promoter activity (Figure 3A). The co-transfection of the GATA1 transcription factor with the MGPmut2 promotor construct resulted in a decrease in luciferase activity of 1.36-fold compared with the mutant construct co-transfected with the empty vector, although it is not a statistically significant reduction in luciferase activity (Figure 3B).

#### 2.2.3. C/EBPα Has a Repressive Effect on the *MGP* Promoter

**Figure 4:**
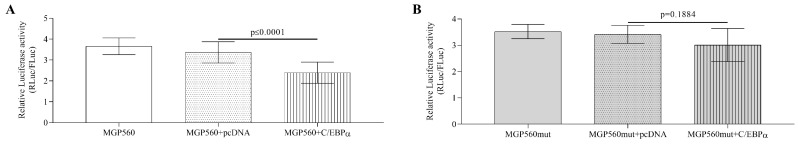
Effect of C/EBPα transcription factor in *MGP* promoter activity. The *MGP* promoter fragment MGP560 (**A**) and the construct MGP560Mut1 (**B**) were co-transfected with the transcription factor C/EBPα. Data are presented as mean ± SD of at least five independent experiments. Statistical significance was determined by a one-way ANOVA statistical test with Tukey’s correction; *p*-values were considered significant when *p* ≤ 0.05.

Co-transfection of the expressing vector carrying the C/EBPα and the MGP560 construct resulted in a significant decrease in the luciferase activity of 1.4-fold (Figure 4A).

To further analyze the repression of the *MGP* promoter by C/EBPα, we transiently co-transfected the MGP560mut1 construct, which contains a mutation at site A, with either an empty vector (pcDNA) or pcDNA-C/EBPα. The results showed that the relative luciferase activity was unaffected (Figure 4B).

#### 2.2.4. Repression in the *MGP* Promoter Is Enhanced by YY1

**Figure 5:**
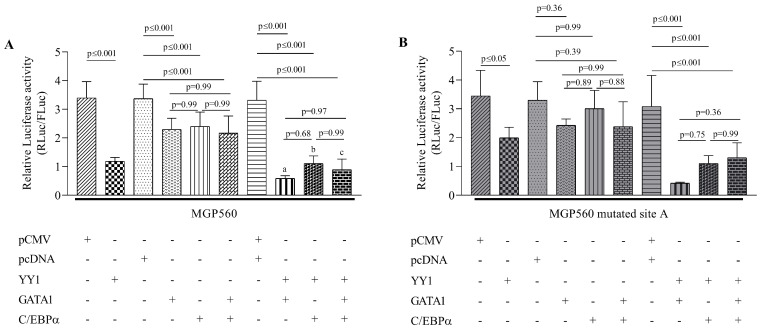
Combinatory interactions of YY1, GATA1, and C/EBPα in the repression of the *MGP* gene. (**A**) Luciferase reporter assays after co-transfection of YY1, GATA1, and C/EBPα expression vectors along with reporter construct MGP560. Repression of promoter activity was observed when either YY1, GATA1, or C/EBPα was overexpressed in the cell culture system. However, simultaneous overexpression of YY1 and GATA1, YY1 and C/EBPα, and YY1, GATA1, and C/EBPα dramatically enhanced the intensity of repression of luciferase activity. Small letters denote the statistical difference between (a) MGP560 with GATA1 and YY1 vs. MGP560 with GATA1 (*p* ≤ 0.001); (b) MGP560 with C/EBPα and YY1 vs. MGP560 with C/EBPα (*p* ≤ 0.001); and (c) MGP560 with C/EBPα, GATA1, and YY1 vs. MGP560 with C/EBPα and GATA1 (*p* ≤ 0.001). (**B**) Mutation analysis of *MGP* promoter using luciferase reporter assays. The binding site for YY1, GATA1, and C/EBPα was mutated in the MGP560 reporter construct and then the construct was co-transfected along with the YY1, GATA1, and C/EBPα expression vectors. The repression in the luciferase activity observed with the MGP560mut2 construct was not compromised with the mutation in the binding site when YY1 was present. Error bars represent ± SD of at least five independent experiments. Statistical significance was determined by a one-way ANOVA statistical test with Tukey’s correction; *p*-values were considered significant when *p* ≤ 0.05.

We employed a series of co-transfection assays with the *MGP* promoter alone or combined with the expression vectors for YY1, GATA1, and C/EBPα TFs to test the possible combined involvement of these factors in the observed response in the *MGP* promoter. Co-transfection of either YY1, GATA1, or C/EBPα along with the MGP560 construct resulted in significant repression in the promoter activity (Figure 5A). However, the level of repression was dramatically affected when YY1 and GATA1, YY1 and C/EBPα, or YY1, GATA1, and C/EBPα were co-transfected with the MGP560 construct, causing 3.9-, 2.2-, and 2.4-fold repression of luciferase activity, respectively, compared to the effect observed with these TFs in the absence of YY1, as shown in Figure 5A.

To further verify our findings about the synergistic action of these TFs, we performed co-transfections with the MGP650mut2 construct. The results showed a repression of the promoter activity in the presence of YY1 (Figure 5B), suggesting that the repression due to YY1 is independent of the binding site identified in the *MGP* promoter and that YY1 may interact with the two TFs to repress *MGP* promoter.

These data suggest that YY1 specifically targets GATA1- and C/EBPα-dependent transcription, although they do not rule out other possibilities.

#### 2.2.5. RUNX2 Enhances *MGP* Promoter Activity

**Figure 6:**
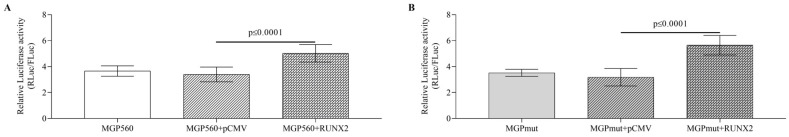
Effect of the RUNX2 transcription factor in *MGP* promoter activity. The *MGP* promoter fragment MGP560 (**A**) and the construct MGP560Mut1 (**B**) were co-transfected with the transcription factor RUNX2 expressing vector. Data are presented as mean ± SD of at least five independent experiments. Statistical significance was determined by a one-way ANOVA statistical test with Tukey correction; *p*-values were considered significant when *p* ≤ 0.05.

In our previous work, we showed a positive correlation between the expression of *RUNX2* mRNA in colorectal cancer (CRC) tissue samples and *MGP* expression; therefore, we wanted to evaluate if RUNX2 affected *MGP* transcription regulation. Co-transfection of the expression vector carrying the RUNX2 triggered a significant increase of 1.5-fold over the control in the luciferase activity of the MGP560 construct (Figure 6A). Co-transfection of RUNX2 with the MGP560mut1 construct still promoted an enhancement in *MGP* promoter activity of 1.8-fold over the control (Figure 6B).

### 2.3. Transcription Factors YY1, GATA1, and C/EBPα May Negatively Regulate MGP Expression in Cancer

**Figure 7:**
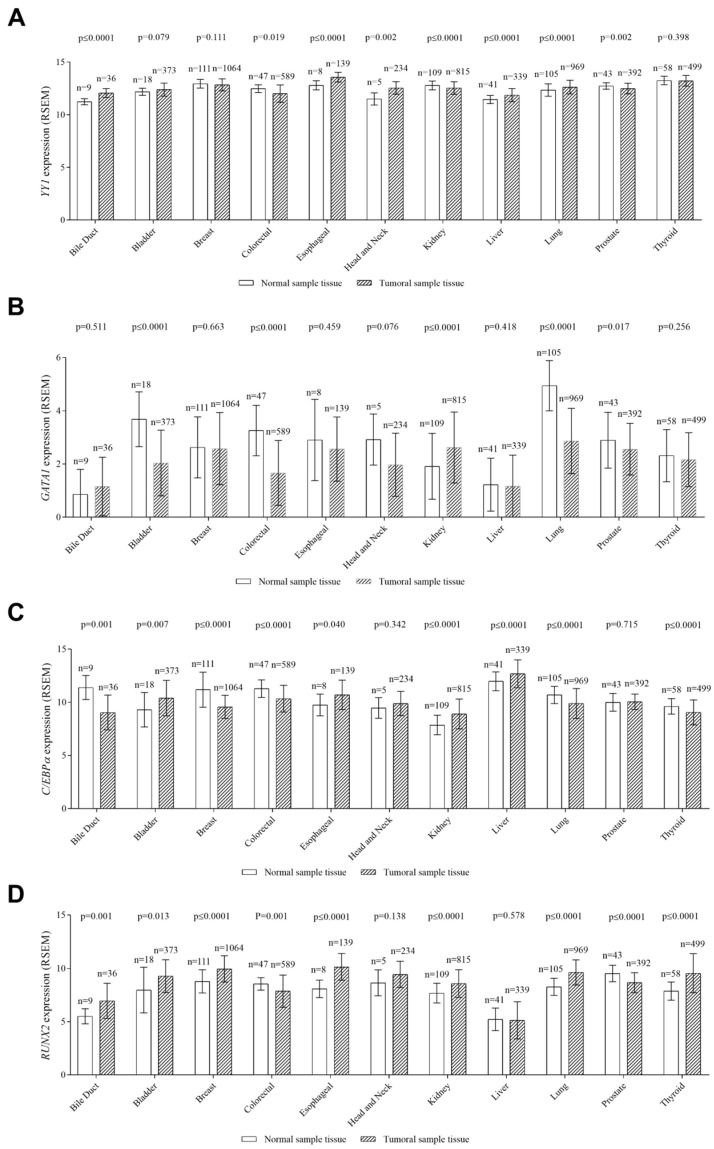
Analysis of *YY1*, *GATA1*, *C/EBPα*, and *RUNX2* gene expression in normal and tumoral samples from TCGA databases. (**A**) *YY1* was upregulated in the cancerous tissue compared to the normal tissue, with the exception of colorectal (*p* = 0.019), kidney (*p* ≤ 0.0001), and prostate (*p* = 0.002), which presented a significant upregulation in the normal tissue. (**B**) *GATA1* was upregulated in the normal tissue compared to the corresponding tumor tissue, except for kidney tissue (*p* ≤ 0.0001), which presented a significant upregulation in the tumor tissue. (**C**) *C/EBPα* presented a significant upregulation in the normal tissue, with the exception of bladder (*p* = 0.007), esophageal (*p* = 0.040), kidney (*p* ≤ 0.0001), and liver (*p* ≤ 0.0001), when compared with the tumoral tissue. (**D**) *RUNX2* was upregulated in the tumoral tissue, contrary to colorectal (*p* = 0.001) and prostate (*p* ≤ 0.0001) cancers, whose expression was upregulated in the normal tissue. Mean ± SD; Mann–Whitney statistic test; the *p*-value was considered statistically different (*p* ≤ 0.05).

We subsequently asked whether the pattern of *MGP* regulation by YY1, GATA1, C/EBPα, and RUNX2 from our in vitro analysis may also be observed in primary clinical data. These factors were significantly up- or downregulated in different cancer tissues relative to noncancerous tissue (Figure 7A–D).

Data from the TCGA database (http://xena.ucsc.edu/), (accessed on 1 September 2022) using unpaired samples of cancer and noncancerous tissues, indicated that *YY1* mRNA levels are significantly upregulated in various types of malignancies, including bile duct, esophageal, head and neck, lung, and liver cancers (Figure 7A). Next, we performed an in silico analysis and, by doing so, found that *YY1* and *MGP* expression exhibited a significant inverse correlation in the bladder and esophageal cancers (Supplementary Figure S1B,E). Hence, these data indicate that YY1 may serve as a negative regulator of *MGP* and that it may play oncogenic roles in different cancers.

*GATA1* expression was significantly higher in noncancerous tissue than in kidney cancer (Figure 7B). *MGP* expression was positively closely related to *GATA1* in bladder, breast, colorectal, esophageal, head and neck, kidney, liver, lung, prostate, and thyroid cancers (Supplementary Figure S1J–S).

The expression of *C/EBPα* was significantly higher in noncancerous tissue than in bile duct, breast, lung, and thyroid cancers, and it was significantly upregulated in bladder, esophageal, kidney, and liver cancerous tissues (Figure 7C). *C/EBPα* and *MGP* expression exhibited a significant inverse correlation in bile duct, bladder, and esophageal cancers and a positive correlation in breast, colorectal, lung, and thyroid cancers (Supplementary Figure S1T–Z).

*RUNX2* was overexpressed in bile duct, bladder, breast, esophageal, kidney, lung, and thyroid cancerous tissues and significantly downregulated in colorectal and prostate cancerous tissues (Figure 7D). *RUNX2* and *MGP* expression exhibited a significant inverse correlation in bladder and lung cancers and a positive correlation in the bile duct and head and neck cancers (Supplementary Figure S1A’–I’).

Gene expression can be influenced by numerous factors, both genetic and environmental. In this context, *MGP* expression was used as the dependent variable in a multiple linear regression model to predict which factors contribute to the deregulation of *MGP* gene expression in these tumors (Table 1). The analysis shows that multiple factors, combined with the expression of transcription factors, significantly modulate *MGP* gene expression in most tumors (Table 1).

### 2.4. Analysis of MGP Gene Mutations and Copy Number Variations in Cancer

**Figure 8:**
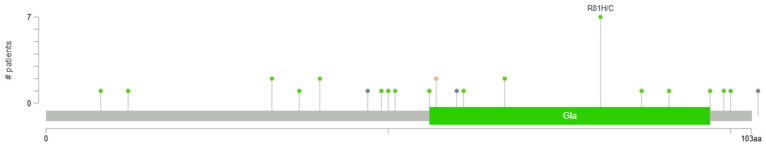
Analysis of a PanCancer study with whole genome data revealing the sites of *MGP* mutations. Light green indicates the missense mutations, gray indicates the truncating mutations, and yellow indicates splice mutations. Gla: Gla domain.

*MGP* gene alterations were studied using the cBioPortal database utilizing the TCGA PanCancer Atlas set (Figure 8 and Table 2). Of the 11 tumors queried, the *MGP* gene was altered in bladder (2%), breast (2%), colorectal (1%), esophageal (4%), head and neck (<1%), liver (<1%), lung (1%), and prostate (2%) cancer tissues (Figures S3–S13), while the somatic mutation frequency was 0.1% for breast and lung; 0.2% for head and neck and prostate; 0.5% for bladder and esophageal; and 0.9% for colorectal cancer tissues (Figures S3–S13). In all the tumors analyzed, the missense variants dominated (Table 2). Then, the association between the *MGP* expression level and mutation as well as copy number alteration status was evaluated for the 11 tumors (Figures S3–S13).

## 3. Discussion

Epigenetic events are progressively being more associated with cancer, being identified as a possible cause for the activation or silencing of certain genes depending on their biological context [16], More recently, it has been published that the downregulation of *MGP* in breast cancer could be due to one methylation site in the proximal region of the *MGP* promotor and higher levels of *MGP* could predict a better survival outcome [17]. Given our previous analysis of the methylation levels in the different types of tumors and their association with *MGP* expression [18], our interest was to explore how the CpG sites found in the promoter region could affect *MGP* transcription.

Transcription factors regulate gene expression to exert its specified function. This study utilized an in silico approach to search for TFs that putatively bind to the CpG sites identified in the *MGP* promoter region and found that YY1, GATA1, and C/EBPα overlap the cg00431549 located at −238bp from the TSS. Our studies suggest that these TFs and RUNX2 may be involved in the regulation of *MGP* expression; of these, GATA1 and C/EBPα appear to be capable of interacting with the CpG site within the *MGP* promoter.

The regulation of *MGP* expression is likely multifactorial involving many transcription factors with activator or repressor functions responding to distinct signaling pathways. Transcription factor YY1 (Yin Yang 1) belongs to the family of “Zinc-Fingers” proteins called GLI-Kruppel [19]. This transcription factor can interact in promoter regions of different genes acting either as an inhibitor or activator of gene expression, thus performing contrary and versatile functions, depending on external factors [20,21,22]. Other studies have shown that YY1 can also exert transcriptional changes through protein–protein interaction [23]. To determine if *MGP* repression seen in the reporter assays is the result of YY1′s direct binding to the *MGP* promoter, electrophoretic mobility shift assays (EMSAs) or ChIP should be performed.

Our results are in concordance with results recently published [22] that showed, using TCGA and GEO datasets, that YY1 was expressed at high levels in most malignancies, and its level of expression was statistically associated with the prognosis of tumor patients.

GATA1 has two highly conserved zinc-finger domains, with one located at the C-terminal responsible for the binding to typical elements (recognizes (A/T)GATA(A/G) motifs) in target gene promoters [24]. GATA1’s first reports indicated a critical function in the formation of early eosinophil precursors and in the differentiation of committed erythroid precursors and megakaryocytes [25]. Emerging evidence indicates that GATA1 is activated in several tumors and is involved in cell growth, apoptosis, tumorigenesis, and aggressiveness of solid tumors. In pancreatic ductal adenocarcinoma tissues, GATA1 was found to be highly expressed and an independent predictor of prognosis and response to gemcitabine therapy through the anti-apoptotic pathway [26]. In CRC, GATA1 was upregulated and associated with a predicted poor clinical outcome [27]. In breast cancer, GATA1 was found to be overexpressed, and it was demonstrated to promote survivin expression [28]. Furthermore, it was shown that GATA1 promotes breast cancer growth and metastasis by regulating VEGF expression [29]. In glioblastomas, it was demonstrated that the interaction of GATA1 and MMP-2 enhanced glioblastoma invasion and migration [30]. It was also suggested that in ovarian cancer, GATA1-regulated JAG1 plays a key regulatory role in cancer cell proliferation and metastasis. On the other hand, it was shown that decreased mRNA expression of GATA1 was associated with tumor aggressiveness and poor outcome in clear cell renal cell carcinoma (ccRCC) [31], implying that GATA1 may be associated with the progression and aggressiveness of ccRCC.

Our results are in concordance with results recently published [32] that compared the mRNA expression levels of the *GATA* family genes in different types of cancers and normal tissue samples using the Oncomine database and showed that *GATA1* was expressed at relatively lower levels in most kinds of cancers than in normal tissues [32]. However, the data in TCGA suggested that the *GATA1* expression was positively correlated with the *MGP* expression in cancer tissues. Further in vivo and in vitro studies are needed to better understand the association of GATA1 and MGP and their role in the different tumors.

We report hereby that YY1 and GATA1 act synergistically, coordinating the repression of *MGP*. It appears that this synergistic action only requires the presence of the corresponding binding site for GATA1 in the *MGP* regulatory region. In fact, when co-transfected with the promoter fragment of the *MGP* gene in the breast cancer cell line MCF-7, it was possible to observe repression in the luciferase activity by the transcription factors GATA1 and C/EBPα, whereas, with the YY1 transcription factor, it was not possible to observe a significant difference in the luciferase activity (Supplementary Figure S2), suggesting that the regulation of gene expression can occur either synergistically or cooperatively depending on the cell type and that the TFs responsible for this regulation can co-occupy regulatory regions of common target genes. The TFs that are involved in synergistic or cooperative gene regulation are often observed to be interacting directly with each other [33]. Whether YY1 and GATA1 directly interact after their binding to the *MGP* gene promoter remains to be determined.

The CCAAT/enhancer binding protein (C/EBPα) is a member of the leucine zipper family of transcription factors [34]. The association between C/EBPα and cancer has been well documented in acute myeloid leukemia (AML) [35,36]. Dysregulation of C/EBPα expression has been found in hemato-lymphoid malignancies including AML. Reduced expression of *C/EBPα* has been observed in lung, breast, and head and neck cancers through epigenetic mechanisms including loss of heterozygosity and DNA methylation [37,38,39,40]. In gastric cancer, *C/EBPα* expression was shown to be downregulated [41]. In ovarian cancer [42] and hepatocellular carcinomas [43], an upregulation of *C/EBPα* mRNA expression was associated with worsening outcomes. Also, in ccRCC *C/EBP*α, mRNA expression was upregulated in TCGA data [44]. Altogether, these results show that, in agreement with our data obtained using the TCGA database, *C/EBPα* expression was significantly upregulated in some cancer types and downregulated in others. This dysregulation is likely to be associated with poor clinical outcomes in cancer. Our in vitro results show that *C/EBPα* seems to bind the *MGP* promoter in a specific binding site, repressing its activity, and our in silico analysis using TCGA data shows that in some tumors, *C/EBPα* and *MGP* expression exhibited a significant inverse correlation, while in others there was a positive correlation.

The transcriptional and post-transcriptional regulations of *MGP* expression are complex [45], and it is therefore possible that downstream of YY1, GATA1, and C/EBPα’s downregulation of *MGP* transcription, other factors are significantly involved in determining the ultimate expression of *MGP* and the corresponding clinical sequelae. It is also important to note that while our work demonstrates robust *MGP* promoter activity reduction via reporter assay, the extent to which these TF-mediated reductions in *MGP* expression result in increased apoptosis, alterations in cell cycle progression, or modulation of other hallmarks of cancer progression is currently under investigation in our laboratory.

Another transcription factor that was shown to be able to regulate *MGP* gene expression was Runt-related transcription factor 2 (RUNX2). In mice, the parathyroid hormone regulates *Mgp* expression through the transcription factors Sp and Runx2 [46]. In addition, it has been demonstrated that *MGP* is regulated by Runx2 in *Xenopus laevis* [47]. Interestingly, in this model, two functional promoters (proximal and distal) were identified in the *MGP* gene, and both were shown to be regulated by Runx2. It was evidenced that co-transfection of ATF4, RUNX2, and SATB2 enhanced the *MGP* human promoter activity in ATDC5 cells [48]. Recently, we showed that *MGP* and *RUNX2* were overexpressed in CRC tissue samples and there was a positive correlation between the two expressions in tumor mucosa [49]. Our current results suggest that RUNX2 binds to the *MGP* promoter and enhances its transcription, as shown through co-transfection experiments. Also, our in silico analysis showed that *RUNX2* was overexpressed in breast, esophageal, kidney, lung, and thyroid cancerous tissues and significantly downregulated in liver and prostate cancerous tissues and that *RUNX2* and *MGP* expression exhibited a significant inverse correlation in bladder and lung cancers and a positive correlation in bile duct and head and neck cancers.

Whether the alterations in the TFs’ expression and/or disturbance in *MGP* methylation are implicated in cancer should be further investigated by comparing the expression of these TFs and the levels of CpG dinucleotide methylation in the *MGP* gene of individuals with different types of cancers compared to the controls.

Investigating gene alterations can provide valuable insights into gene function and its role in cancer development and progression. Using cBioPortal, we examined the frequency and distribution of alterations across the *MGP* gene. The alteration frequency was relatively low across various cancer samples (generally less than or around 4%), with missense variants being the most common. Overall, the observed mutations and copy number alterations did not appear to significantly affect the mRNA expression z-scores compared to normal samples. Regarding the transcription factors analyzed, some studies have reported varying percentages of mutations associated with different types of tumors. For example, the genetic variants of the *RUNX2* gene in cancer patients were recently reviewed by Lin T-C [50].

Our discovery of novel transcriptional repressors of *MGP* may provide new insights into its altered expression in a significant subset of cancers. We also provide evidence for a possible role of YY1, GATA1, and C/EBPα in *MGP* transcription regulation in human cancers. These efforts may help to further determine the clinical significance of MGP across different types of cancer. However, further verification experiments are necessary to confirm the results of these analyses.

Although at present no MGP- or RUNX2-specific inhibitors have been approved for cancer treatment, these molecules are under active investigation as potential therapeutic targets.

## 4. Material and Methods

### 4.1. CpG Site Identification in the MGP Gene

The identification of CpG nucleotides in the *MGP* gene was performed by analyzing USCS genome browser data (https://genome.ucsc.edu/) (accessed on 12 November 2018) [51]. This public database is available online and allows for the visualization of CpG ID probes for specific CpG sites within a given gene, generated by the Illumina Infinium Human Methylation 450 Bead Array platform.

### 4.2. In Silico Analysis of MGP Promoter

A human *MGP* promoter sequence spanning from −527 to +48 (from now on referred to as MGP560), related to the beginning of the transcriptional start site, was retrieved from the NCBI database (www.ncbi.nlm.nih.gov, NCBI Reference Sequence: NG_023331.1).

Evaluation for putative transcription factors binding sites (TFBSs) was carried out through the analysis of three online bioinformatics tools: (i) PROMO (https://alggen.lsi.upc.es/cgi-bin/promo_v3/promo/promoinit.cgi?dirDB=TF_8.3) (accessed on 6 January 2020) [52,53]; (ii) TF BIND (https://tfbind.hgc.jp/) (accessed on 6 January 2020) [54]; and (iii) LASAGNA—search 2.0 (http://biogrid.engr.uconn.edu/lasagna_search/) (accessed on 6 January 2020) [55]. The transcription factors predicted in at least two of the software analyzed were selected for further in vitro validation.

### 4.3. Luciferase Reporter Constructs of MGP Promoter

The *MGP* promoter fragment (MGP560) inserted into pGL2 described previously by Kirfel et al. 1997 [7] was removed and inserted into *Bgl*II—*Hind*III restriction sites of the reporter plasmid pGL3–Basic (Promega, Madison, WI, USA) and sequenced in both strands to confirm the correct orientation and insertion into the plasmid.

### 4.4. Site-Directed Mutagenesis

For the evaluation of the effect of the selected TFs and their binding to the CpG sites in the *MGP* promoter, the MGP560 luciferase construct was used as a DNA template to generate six luciferase constructs, each containing site-directed mutations for the following: the CpG site cg00431549 (MGP560mut1); a mutation abolishing the entire core sequence for YY1 (MGP560mut2); the second putative binding site for the YY1 transcription factor (MGP560mut3); the third putative binding site of YY1 located at the transcription start site (TSS) (MGP560mut4); both mutations for the CpG site and the second putative binding site for YY1 (MGP560mut5); and both mutations abolishing the entire YY1 core sequence and the third putative binding site for YY1 (MGP560mut6). These constructs were generated using the QuikChange Lightning Site-Directed Mutagenesis kit (Agilent Technologies, Santa Clara, CA, USA) following the manufacturer’s instructions and the specific primers described in Table 3 (the inserted mutations are depicted in bold and underlined).

### 4.5. Transcription Factor Expressing Vectors

The expression plasmid containing the human YY1 transcription factor was a kind gift from Dr. Yang Shi (Harvard Medical School, pCMV-hYY1). The expression plasmid containing the human GATA1 transcription factor was purchased from Addgene (catalog number # 118352, Watertown, MA, USA). The expression plasmid containing the C/EBPα transcription factor was a kind gift from Dr. Pierre Fafournoux (French National Institute for Agriculture, Food, and Environment INRAE, Unité de Nutrition Humaine, France, pcDNA-C/EBPα). The expression plasmid containing the mouse full-length Runx2 type II isoform was a kind gift of Dr. Gerard Karsenty (Baylor College of Medicine, Houston, TX, USA, pCMV-Osf2/Cbfa1).

### 4.6. Cell Culture

Human embryonic kidney cells (HEK-293) and MCF-7 were cultured in Dulbecco’s modified Eagle’s medium (DMEM, Invitrogen, Carlsbad, CA, USA) supplemented with 10% (*v*/*v*) of fetal bovine serum (FBS, Invitrogen), 1% (*v*/*v*) of L-glutamine (200 mM, Invitrogen), and 1% of penicillin–streptomycin (10.000 U/mL). The cells were maintained at 37 °C in a 5% CO_2_ humidified atmosphere and subdivided every 3 days.

### 4.7. Luciferase Reporter Assays

One day before the transfections, HEK-293 and MCF-7 cells were seeded in a 24-well plate at the density of 5 × 10^4^ cells per well. After reaching a confluence of 50–60%, 250 ng of the *MGP* promoter pGL3-luciferase construct (MGP560) or the *MGP* promoter fragments containing the site-directed mutations as described in Section 2.4 was co-transfected with 25 ng of each expression plasmid containing the transcription factors or empty expression plasmids with 0.5 µL of XtremeGENE HP DNA transfection reagent (Roche, Basel, Switzerland) and 25 ng of the pRL–TK vector as the internal control per well.

The pGL3-Basic and pGL3-control vectors were used as negative and positive controls to monitor transfection efficiency. After 48 h post-transfection, cells were lysed, and firefly and renilla activities were measured with a Dual-Luciferase Reporter Assay Kit (Promega) according to the manufacturer’s instructions, using a multiplate reader (BioTek Synergy 4, BioTek, Winooski, VT, USA). The relative luciferase activity was calculated based on the ratio from firefly and renilla luciferase activities, and independent experiments were performed at least five times with duplicates per well.

### 4.8. TCGA Data Analysis

Cohort studies from different types of cancer were accessed through The Cancer Genome Atlas (TCGA) database via the Cancer Genome Browser (http://xena.ucsc.edu/, accessed 1 September 2022), with data originally published by the National Cancer Institute. Data including gene expression were downloaded from the UCSC Xena browser (TCGA) data portal (https://xenabrowser.net/, accessed 1 September 2022). All the downloaded data involved in this study were downloaded from TCGA using TCGA publication guidelines and data access policies; thus, additional approval by the local Ethics Committee was not needed. mRNA expression and clinical data from the TCGA database were used to assess correlations between the expression of *MGP* and the expression of the transcription factors analyzed (*YY1*, *GATA1*, *C/EBPα*, and *RUNX2*).

### 4.9. Gene Mutation and Copy Number Alteration Analysis

The MGP mutations and their location were detected using cBioPortal (http://www.cbioportal.org/; accessed on 15 November 2024) [56,57]. The web tool facilitated the generation of graphical visualizations for mutation and copy number alterations in the selected cancer dataset using the Oncoprint and MutationMapper modules. The dataset chosen for visualization was the TCGA PanCancer Atlas, which included selected mutations, structural variants, potential copy number alterations identified by GISTIC, and mRNA expression genomic profiles.

### 4.10. Statistical Analysis

The results are presented as mean ± standard deviation (SD) and the statistical analysis was performed using Prism 8 (GraphPad Software, Boston, MA, USA) and the SPSS software program version 26 (IBM, 2010, Chicago, IL, USA). Comparisons between gene expression were estimated using non-parametric statistical tests: Mann–Whitney U and Kruskal–Wallis. Significant differences were determined by a one-way ANOVA statistical test, with Tukey’s post-test correction, where a two-sided *p*-value less than 0.05 was demarcated as statistically significant (*p* ≤ 0.05).

The correlation between the expression of *MGP* and the TFs’ expression was verified by Spearman rank correlation analysis.

A multiple linear regression model was used to predict significant factors that might be influencing *MGP* gene expression (dependent variable). As for the explanatory variables for the regression model, we selected the age, gender, tumor stage, the pathologic T, N, and M stages, and the gene expression of transcription factors *YY1*, *GATA1*, *C/EBPα*, and *RUNX2*.

## Figures and Tables

**Table 1:** Multiple linear regression analysis of *MGP* gene expression.

	Bile Duct							Bladder						
Explanatory Variables	Unstandardized Coefficient		Standardized Coefficient	t	*p* Value	R^2^ of the Model	*p* Value	Unstandardized Coefficient		Standardized Coefficient	t	*p* Value	R^2^ of the Model	*p* Value
	**B**	**S.D**	**Beta**			0.691	**0.0001**	**B**	**S.D**	**Beta**			0.325	**0.0001**
MGP (dependent variable)	−5.299	5.017		−1.056	0.298			11.511	2.029		5.673	**0.0001**		
Age	0.004	0.16	0.029	0.257	0.799			0.021	0.009	0.104	2.4	**0.017**		
Gender	−0.362	0.362	−0.102	−1.001	0.324			−0.270	0.202	−0.058	−1.340	0.181		
Tumor Stage	−0.667	0.281	−0.604	−2.372	**0.024**			0.130	0.197	0.052	0.66	0.51		
Pathological M	0.992	0.559	0.261	1.776	0.085			−0.060	0.17	−0.016	−0.352	0.725		
Pathological N	0.47	0.476	0.136	0.987	0.331			0.234	0.143	0.106	1.644	0.101		
Pathological T	0.664	0.23	0.546	2.88	**0.007**			0.106	0.056	0.111	1.906	0.057		
YY1	1.144	0.424	0.332	2.695	**0.011**			−0.321	0.156	−0.094	−2.062	**0.04**		
GATA1	−0.486	0.191	−0.287	−2.541	**0.016**			0.592	0.072	0.362	8.253	**0.0001**		
CEBPα	−0.291	0.111	−0.292	−2.621	**0.013**			−0.181	0.055	−0.146	−3.301	**0.001**		
RUNX2	0.579	0.14	0.519	4.119	**0.0001**			0.213	0.06	0.163	3.552	**0.0001**		
	**Breast**							**Colorectal**						
**Explanatory Variables**	**Unstandardized Coefficient**		**Standardized Coefficient**	**t**	***p *** **Value**	**R^2^ of the Model**	***p *** **Value**	**Unstandardized Coefficient**		**Standardized Coefficient**	**t**	***p *** **Value**	**R^2^ of the Model**	***p *** **Value**
	**B**	**S.D**	**Beta**			0.046	**0.0001**	**B**	**S.D**	**Beta**			0.484	**0.0001**
MGP (dependent variable)	6.501	1.837		3.539	**0.0001**			−0.861	1.039		−0.829	0.408		
Age	0.019	0.005	0.121	4.166	**0.0001**			−0.002	0.004	−0.013	−0.450	0.653		
Gender	1.368	0.555	0.071	2.467	**0.014**			0.147	0.109	0.039	1.346	0.179		
Tumor Stage	−0.080	0.043	−0.098	−1.865	0.062			0.014	0.026	−0.028	−0.553	0.58		
Pathological M	0.355	0.147	0.071	2.407	**0.016**			0.093	0.107	0.028	0.87	0.385		
Pathological N	0.031	0.023	0.059	1.374	0.17			0.083	0.038	0.092	2.182	**0.029**		
Pathological T	0.01	0.042	0.009	0.23	0.81			0.182	0.08	0.079	2.281	**0.023**		
YY1	0.258	0.11	0.07	2.346	**0.019**			0.251	0.106	0.107	2.353	**0.019**		
GATA1	0.117	0.045	0.077	2.585	**0.01**			0.277	0.046	0.186	5.996	**0.0001**		
CEBPα	0.075	0.049	0.46	1.539	0.124			0.154	0.053	0.102	2.923	**0.004**		
RUNX2	0.059	0.048	0.037	1.222	0.222			0.611	0.053	0.478	11.429	**0.0001**		
	**Esophageal**							**Head and Neck**						
**Explanatory Variables**	**Unstandardized Coefficient**		**Standardized Coefficient**	**t**	***p *** **Value**	**R^2^ of the Model**	***p *** **Value**	**Unstandardized Coefficient**		**Standardized Coefficient**	**t**	***p *** **Value**	**R^2^ of the Model**	***p *** **Value**
	**B**	**S.D**	**Beta**			0.341	**0.0001**	**B**	**S.D**	**Beta**			0.358	**0.0001**
MGP (dependent variable)	13.485	3.855		3.498	**0.001**			8.246	1.977		4.172	**0.0001**		
Age	0.017	0.011	0.122	1.63	0.111			0.005	0.008	0.039	0.702	0.483		
Gender	0.07	0.349	0.015	0.201	0.841			−0.607	0.204	−0.163	−2.972	**0.003**		
Tumor Stage	0.089	0.112	0.116	0.793	0.429			0.178	0.157	0.103	1.132	0.259		
Pathological M	−0.505	0.331	−0.147	−1.525	0.13			0.268	0.211	0.07	1.271	0.205		
Pathological N	0.159	0.246	0.075	0.648	0.518			0.02	0.056	0.025	0.36	0.719		
Pathological T	0.156	0.187	0.077	0.834	0.406			−0.032	0.087	−0.029	−0.365	0.715		
YY1	−0.431	0.282	−0.124	−1.529	0.128			−0.414	0.173	−0.153	−2.397	**0.017**		
GATA1	0.532	0.101	0.376	5.258	**0.001**			0.523	0.077	0.374	6.786	**0.0001**		
CEBPα	−0.156	0.094	−0.125	−1.671	0.097			0.072	0.092	0.049	0.776	0.438		
RUNX2	0.268	0.11	0.203	2.44	**0.016**			0.517	0.082	0.385	6.312	**0.0001**		
	**Kidney**							**Liver**						
**Explanatory Variables**	**Unstandardized Coefficient**		**Standardized Coefficient**	**t**	***p *** **Value**	**R^2^ of the model**	***p *** **Value**	**Unstandardized Coefficient**		**Standardized Coefficient**	**t**	***p *** **Value**	**R^2^ of the Model**	***p *** **Value**
	**B**	**S.D**	**Beta**			0.366	**0.0001**	**B**	**S.D**	**Beta**			0.309	**0.0001**
MGP (dependent variable)	−2.521	1.171		−2.154	**0.032**			6.804	1.736		3.92	**0.0001**		
Age	−0.005	0.004	−0.029	−1099	0.272			−0.004	0.006	−0.029	−0.640	0.522		
Gender	0.106	0.109	0.026	0.97	0.332			0.07	0.175	0.018	0.402	0.688		
Tumor Stage	−0.319	0.111	−0.190	−2.879	**0.004**			0.08	0.125	0.065	0.64	0.522		
Pathological M	0.896	0.115	0.268	7.819	**0.0001**			0.049	0.229	0.011	0.217	0.828		
Pathological N	0.084	0.086	0.028	0.972	0.331			0.048	0.218	0.012	0.22	0.826		
Pathological T	0.138	0.04	0.207	3.442	**0.001**			−0.076	0.091	−0.083	−0.834	0.405		
YY1	0.902	0.096	0.276	9.419	**0.001**			−0.112	0.155	−0.037	−0.719	0.472		
GATA1	0.007	0.04	0.005	0.171	0.864			−0.035	0.074	−0.021	−0.467	0.641		
CEBPα	−0.136	0.039	−0.099	−3.450	**0.001**			0.091	0.069	0.063	1.319	0.188		
RUNX2	0.438	0.048	0.297	9.181	**0.0001**			0.63	0.054	0.566	11.605	**0.0001**		
	**Lung**							**Prostate**						
**Explanatory Variables**	**Unstandardized Coefficient**		**Standardized Coefficient**	**t**	***p *** **Value**	**R^2^ of the model**	***p *** **Value**	**Unstandardized Coefficient**		**Standardized Coefficient**	**t**	***p *** **Value**	**R^2^ of the Model**	***p *** **Value**
	**B**	**S.D**	**Beta**			0.31	**0.0001**	**B**	**S.D**	**Beta**			0.244	**0.0001**
MGP (dependent variable)	9.204	0.903		10.195	**0.0001**			7.867	1.319		5.964	**0.0001**		
Age	0.009	0.004	0.055	2.103	**0.036**			0.004	0.007	0.026	0.609	0.543		
Gender	0.384	0.085	0.119	4.519	**0.0001**			-	-	-	-	-		
Tumor Stage	0.007	0.032	0.01	0.207	0.836			-	-	-	-	-		
Pathological M	0.012	0.085	0.004	0.137	0.891			0.216	0.424	0.022	0.51	0.611		
Pathological N	0.02	0.085	0.009	0.239	0.811			0.132	0.131	0.047	1.008	0.314		
Pathological T	−0.072	0.028	−0.085	−2.544	**0.011**			0.19	0.053	0.17	3.591	**0.0001**		
YY1	−0.165	0.074	−0.068	−2.245	**0.025**			0.191	0.116	0.089	1.648	0.1		
GATA1	0.552	0.032	0.471	17.401	**0.0001**			0.289	0.049	0.267	5.913	**0.0001**		
CEBPα	0.102	0.034	0.089	2.993	**0.003**			−0.081	0.07	−0.056	−1.160	0.274		
RUNX2	0.186	0.037	0.143	5.02	**0.0001**			0.298	0.056	0.27	5.341	**0.0001**		
	**Thyroid**													
**Explanatory Variables**	**Unstandardized Coefficient**		**Standardized Coefficient**	**t**	***p *** **Value**	**R^2^ of the Model**	***p *** **Value**							
	**B**	**S.D**	**Beta**			0.146	**0.0001**							
MGP (dependent variable)	7.232	1.252		5.776	**0.0001**									
Age	0.000215	0.003	0.003	0.064	0.949									
Gender	0.114	0.094	0.049	1.205	0.229									
Tumor Stage	−0.074	0.046	−0.097	−1.597	0.111									
Pathological M	0.244	0.08	0.124	3.068	**0.002**									
Pathological N	0.039	0.039	0.047	1.013	0.311									
Pathological T	0.02	0.035	0.027	0.579	0.563									
YY1	0.172	0.092	0.082	1.868	0.062									
GATA1	−0.000286	0.043	−0.000279	−0.007	0.995									
CEBPα	0.231	0.04	0.255	5.779	**0.0001**									
RUNX2	0.152	0.026	0.266	5.907	**0.0001**									

**Table 2:** *MGP* mutations in a PanCancer study of whole genomes.

Cancer Type	Protein Change	Mutation Type	Variant Type	Copy Number	Mutations in Sample	TCGA PanCanAtlas Cancer Type Acronym
Breast Cancer	CPB1-MGP Fusion	fusion	NA	Diploid	39	BRCA
Breast Cancer	CSN1S1-MGP Fusion	fusion	NA	Diploid	5	BRCA
Breast Cancer	MGP-ESR1 Fusion	fusion	NA	Diploid	31	BRCA
Breast Cancer	SCGB2A2-MGP Fusion	fusion	NA	Gain	21	BRCA
Breast Cancer	H4C16-MGP Fusion	fusion	NA	Gain	130	BRCA
Endometrial Cancer	A8P	Missense_Mutation	SNP	Diploid	11,440	UCEC
Breast Cancer	TMEM9B-MGP Fusion	fusion	NA		49	BRCA
Breast Cancer	WBP11-MGP Fusion	fusion	NA	Amp	73	BRCA
Endometrial Cancer	A12T	Missense_Mutation	SNP	Diploid	13,820	UCEC
Endometrial Cancer	P33S	Missense_Mutation	SNP	Diploid	7385	UCEC
Melanoma	P33S	Missense_Mutation	SNP		387	SKCM
Endometrial Cancer	R37M	Missense_Mutation	SNP	Diploid	7793	UCEC
Head and Neck Cancer	A40T	Missense_Mutation	SNP	ShallowDel	116	HNSC
Esophagogastric Cancer	A40V	Missense_Mutation	SNP	Diploid	570	STAD
Bladder Cancer	Q47 *	Nonsense_Mutation	SNP	Diploid	86	BLCA
Non-Small Cell Lung Cancer	R49T	Missense_Mutation	SNP		383	LUAD
Endometrial Cancer	W50L	Missense_Mutation	SNP	Diploid	13,201	UCEC
Endometrial Cancer	R51I	Missense_Mutation	SNP	Diploid	13,852	UCEC
Esophagogastric Cancer	E56A	Missense_Mutation	SNP	Diploid	1305	STAD
Bladder Cancer	X57_splice	Splice_Region	SNP	Gain	159	BLCA
Colorectal Cancer	X57_splice	Splice_Region	SNP	ShallowDel	201	COAD
Glioma	E60 *	Nonsense_Mutation	SNP	Diploid	10,856	LGG
Colorectal Cancer	R61C	Missense_Mutation	SNP	Gain	1089	COAD
Melanoma	E67K	Missense_Mutation	SNP	Gain	1011	SKCM
Colorectal Cancer	E67K	Missense_Mutation	SNP	Diploid	6313	COAD
Endometrial Cancer	R81C	Missense_Mutation	SNP	Diploid	12,979	UCEC
Colorectal Cancer	R81C	Missense_Mutation	SNP	Gain	142	COAD
Colorectal Cancer	R81C	Missense_Mutation	SNP	Gain	103	COAD
Mature B-Cell Neoplasms	R81H	Missense_Mutation	SNP	ShallowDel	154	DLBC
Prostate Cancer	R81H	Missense_Mutation	SNP	Diploid	761	PRAD
Endometrial Cancer	R81H	Missense_Mutation	SNP	Diploid	10,942	UCEC
Endometrial Cancer	R81H	Missense_Mutation	SNP	Diploid	9645	UCEC
Melanoma	G87R	Missense_Mutation	SNP	Diploid	101	SKCM
Ovarian Epithelial Tumor	A91V	Missense_Mutation	SNP	Gain	173	OV
Melanoma	R97K	Missense_Mutation	SNP	Gain	1130	SKCM
Esophagogastric Cancer	R99C	Missense_Mutation	SNP	Diploid	103	ESCA
Melanoma	R100Q	Missense_Mutation	SNP	Diploid	3288	SKCM
Endometrial Cancer	*104Lext*2	Nonstop_Mutation	SNP	Diploid	1740	UCEC

* nonsense mutations.

**Table 3:** Primers used for PCR-based site-directed mutagenesis. Bold uppercase and underlined bases represent mutations concerning the wild-type sequence, in the consensus binding sites for transcription factors.

Name	Orientation	Sequence (5′–3′)
MGP560mut1	sense	5′- CCCAGGTCTGTCCCCAAGCATA**GT**ATGGCCAAAACTT-3′
	antisense	5′-AAGTTTTGGCCAT**AC**TATGCTTGGGGACAGACCTGGG-3′
MGP560mut2	sense	5′-TTTGCCCAGGTCTGTCCCCAAGCATA**GTCCCT**CCAAAACTTCTGCACCAGAGC-3′
	antisense	5′-GCTCTGGTGCAGAAGTTTTGG**AGGGAC**TATGCTTGGGGACAGACCTGGGCAAA-3′
MGP560mut3	sense	5′-GCCCACTCAGAGTAGATAATATC**CCC**AAGGAATGACTGTTTGGGAAAAG-3′
	antisense	5′-CTTTTCCCAAACAGTCATTCCTT**GGG**GATATTATCTACTCTGAGTGGGC-3′
MGP560mut4	sense	5′-TATAAAAACCTCACAGCCTTCCACTAACAT**AATGC**AGGAGCCTCTCTCCCTACTGC-3′
	antisense	5′-GCAGTAGGGAGAGAGGCTCCT**GCATT**ATGTTAGTGGAAGGCTGTGAGGTTTTTATA-3′

## Data Availability

Data is contained within the article and Supplementary Materials.

## Supplementary Materials

The supporting information can be downloaded at https://www.mdpi.com/article/10.3390/ijms252312597/s1.
