# Supplementary material for: Transcriptional Regulation of the Human MGP Promoter: Identification of Downstream Repressors
Source: Int J Mol Sci. 2024 Nov 23;25(23):12597. doi: 10.3390/ijms252312597 (PMC11641715; doi:10.3390/ijms252312597)
Supplement: Supplementary file 1 [file ijms-25-12597-s001.zip › ijms-3256472-supplementary.pdf]

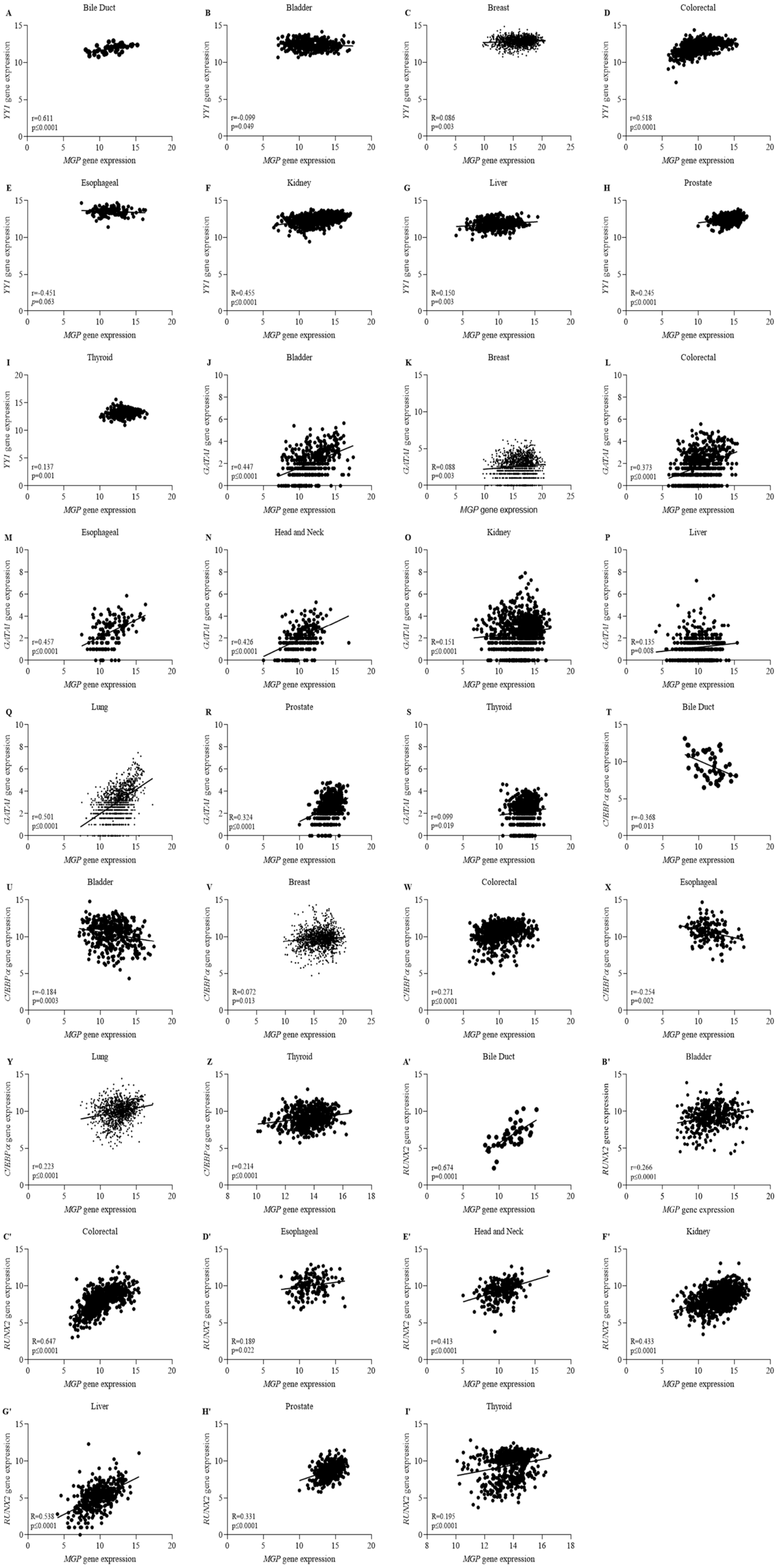
A
Bile Duct
YY1 gene expression
r=0.611
p≤0.0001
MGP gene expression
B
Bladder
YY1 gene expression
r=-0.099
p=0.049
MGP gene expression
C
Breast
YY1 gene expression
R=0.086
p=0.003
MGP gene expression
D
Colorectal
YY1 gene expression
r=0.518
p≤0.0001
MGP gene expression
E
Esophageal
YY1 gene expression
r=-0.451
p=0.063
MGP gene expression
F
Kidney
YY1 gene expression
R=0.455
p≤0.0001
MGP gene expression
G
Liver
YY1 gene expression
R=0.150
p=0.003
MGP gene expression
H
Prostate
YY1 gene expression
R=0.245
p≤0.0001
MGP gene expression
I
Thyroid
YY1 gene expression
r=0.137
p=0.001
MGP gene expression
J
Bladder
GATA1 gene expression
r=0.447
p≤0.0001
MGP gene expression
K
Breast
GATA1 gene expression
R=0.088
p=0.003
MGP gene expression
L
Colorectal
GATA1 gene expression
r=0.373
p≤0.0001
MGP gene expression
M
Esophageal
GATA1 gene expression
r=0.457
p≤0.0001
MGP gene expression
N
Head and Neck
GATA1 gene expression
r=0.426
p≤0.0001
MGP gene expression
O
Kidney
GATA1 gene expression
R=0.151
p≤0.0001
MGP gene expression
P
Liver
GATA1 gene expression
r=0.135
p=0.008
MGP gene expression
Q
Lung
GATA1 gene expression
r=0.501
p≤0.0001
MGP gene expression
R
Prostate
GATA1 gene expression
R=0.324
p≤0.0001
MGP gene expression
S
Thyroid
GATA1 gene expression
r=0.099
p=0.019
MGP gene expression
T
Bile Duct
C/EBPα gene expression
r=-0.368
p=0.013
MGP gene expression
U
Bladder
C/EBPα gene expression
r=-0.184
p=0.0003
MGP gene expression
V
Breast
C/EBPα gene expression
R=0.072
p=0.013
MGP gene expression
W
Colorectal
C/EBPα gene expression
r=0.271
p≤0.0001
MGP gene expression
X
Esophageal
C/EBPα gene expression
r=-0.254
p=0.002
MGP gene expression
Y
Lung
C/EBPα gene expression
r=0.223
p≤0.0001
MGP gene expression
Z
Thyroid
C/EBPα gene expression
r=0.214
p≤0.0001
MGP gene expression
A'
Bile Duct
RUNX2 gene expression
r=0.674
p=0.0001
MGP gene expression
B'
Bladder
RUNX2 gene expression
r=0.266
p≤0.0001
MGP gene expression
C'
Colorectal
RUNX2 gene expression
R=0.647
p≤0.0001
MGP gene expression
D'
Esophageal
RUNX2 gene expression
R=0.189
p=0.022
MGP gene expression
E'
Head and Neck
RUNX2 gene expression
r=0.413
p≤0.0001
MGP gene expression
F'
Kidney
RUNX2 gene expression
R=0.433
p≤0.0001
MGP gene expression
G'
Liver
RUNX2 gene expression
R=0.538
p≤0.0001
MGP gene expression
H'
Prostate
RUNX2 gene expression
R=0.331
p≤0.0001
MGP gene expression
I'
Thyroid
RUNX2 gene expression
R=0.195
p≤0.0001
MGP gene expression

Supplementary Figure S1. Correlation between expression of *MGP* and TFs in cancer. (A–I) *MGP* expression levels were negatively closely related to *YY1* in bladder cancer. (**J–S**) *MGP* expression was positively closely related with *GATA1* in bladder, breast, colorectal, esophageal, head and neck, kidney, liver, lung, prostate, and thyroid cancers. (**T–Z**) *MGP* expression was negatively closely related to *C/EBPα* in bile duct, bladder, and esophageal cancers, and positively closely related to *C/EBPα* in breast, colorectal, lung, and thyroid cancers. (**A'–I'**) *MGP* expression was positively closely related with *RUNX2* in bile duct, bladder, colorectal, esophageal, head and neck, kidney, liver, prostate, and thyroid cancers.

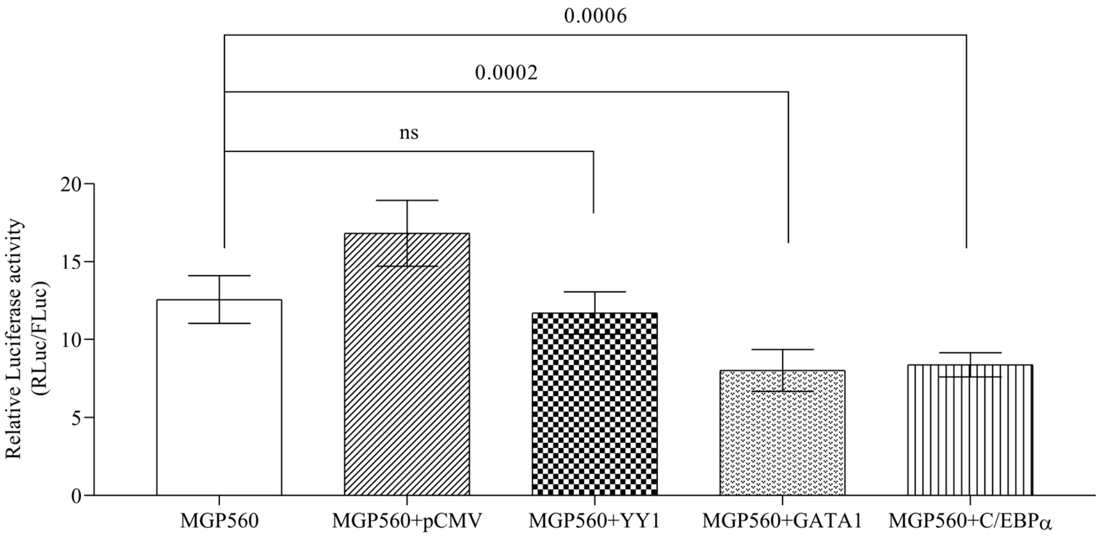

Supplementary Figure S2. Effect of YY1, GATA1, and C/EBPα transcription factors on *MGP* promoter activity. The *MGP* promoter fragment MGP560 was co-transfected with the YY1, GATA1, and C/EBPα expression vectors. Data are presented as mean ± SD of at least five independent experiments. Statistical significance was determined by a one-way ANOVA statistical test with Tukey's correction; *p*-values were considered significant when $p \leq 0.05$.

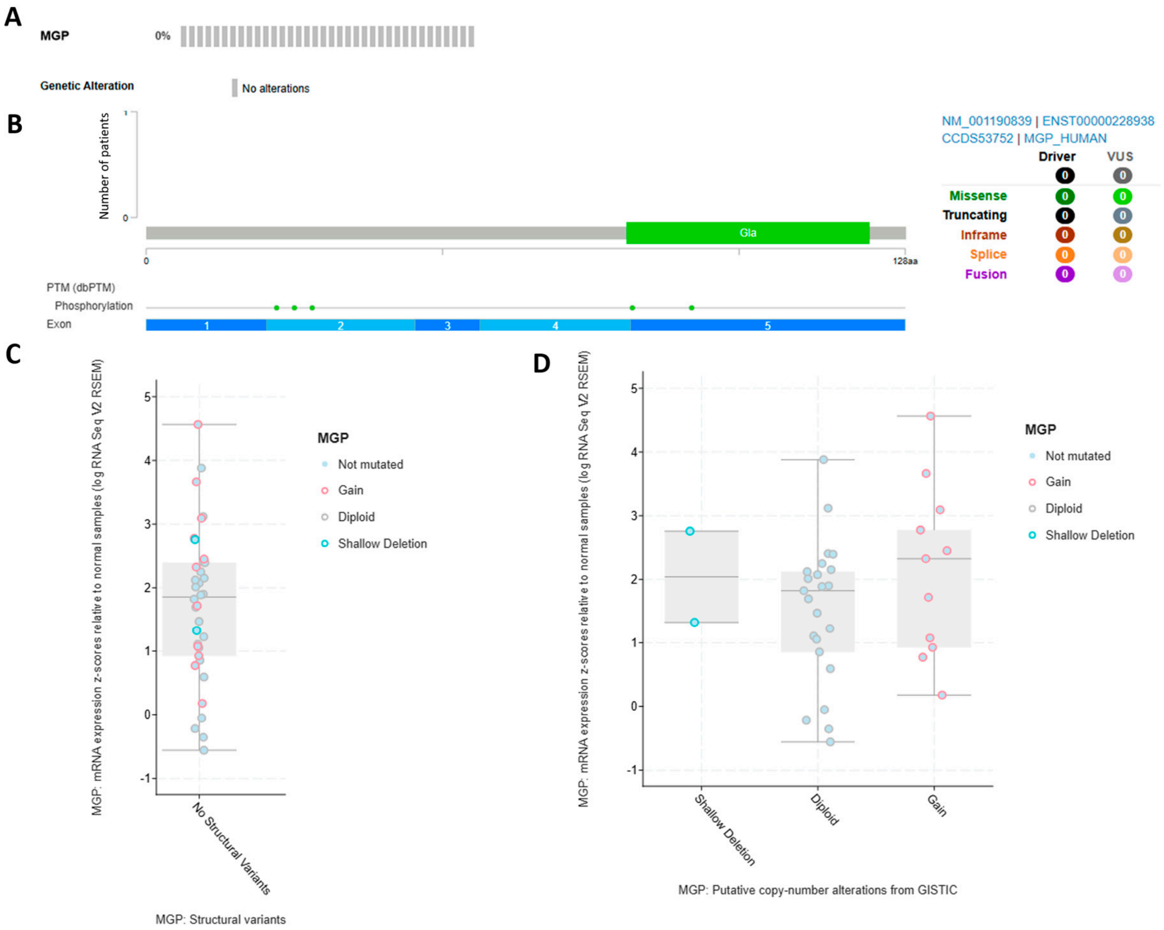

Supplementary Figure S3. The graphical representation and analysis of mutations and copy number alterations in the *MGP* gene for bile duct samples, using cBioPortal. (A) Oncoprint showing the overall genetic alterations. (B) Distribution of mutations and their effect on the protein, exonic regions, and topological structure. (C) Correlation between mutations and mRNA expression z-scores relative to normal samples. (D) Correlation between copy number alterations and mRNA expression z-scores relative to normal samples.

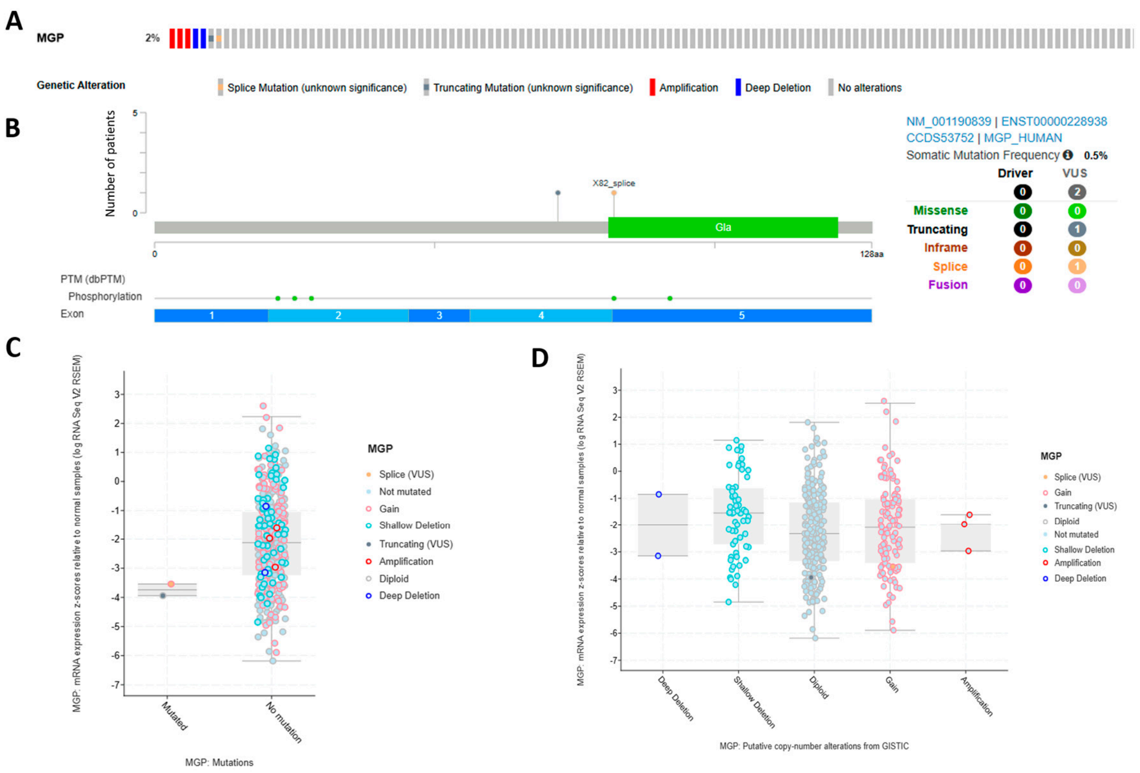

Supplementary Figure S4. Same analysis as Figure S3, but for bladder cancer samples.

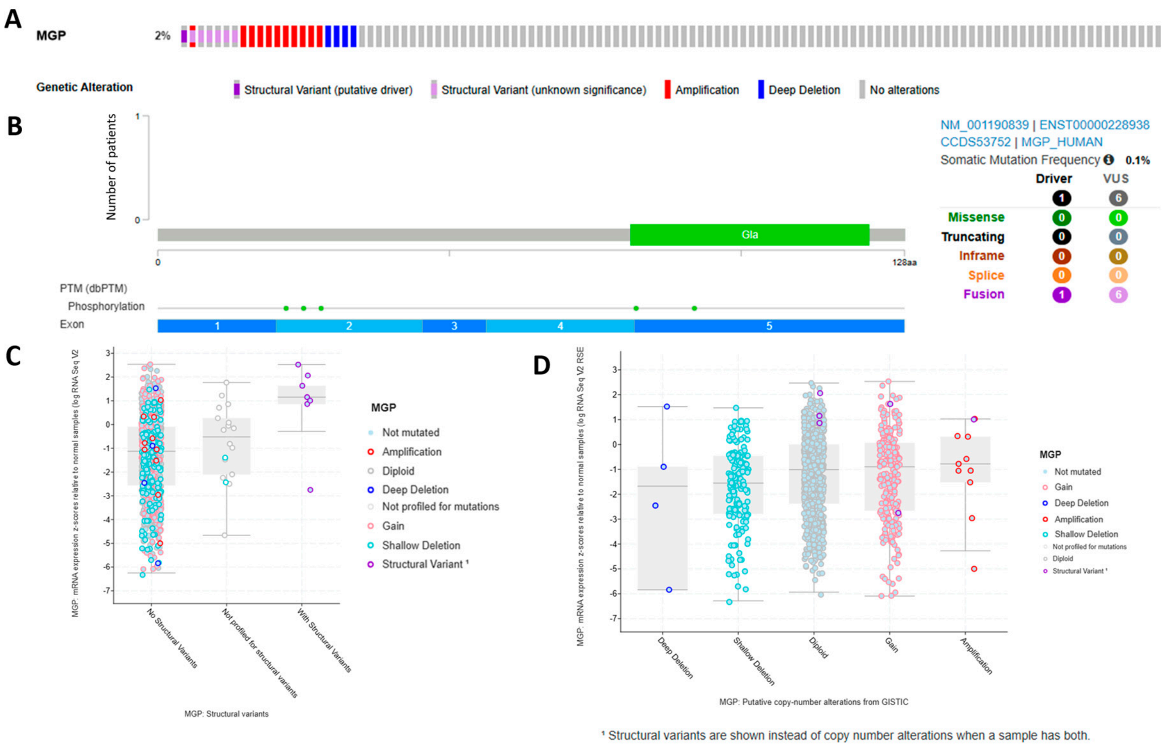

Supplementary Figure S5. Same analysis as Figure S3, but for breast cancer samples.

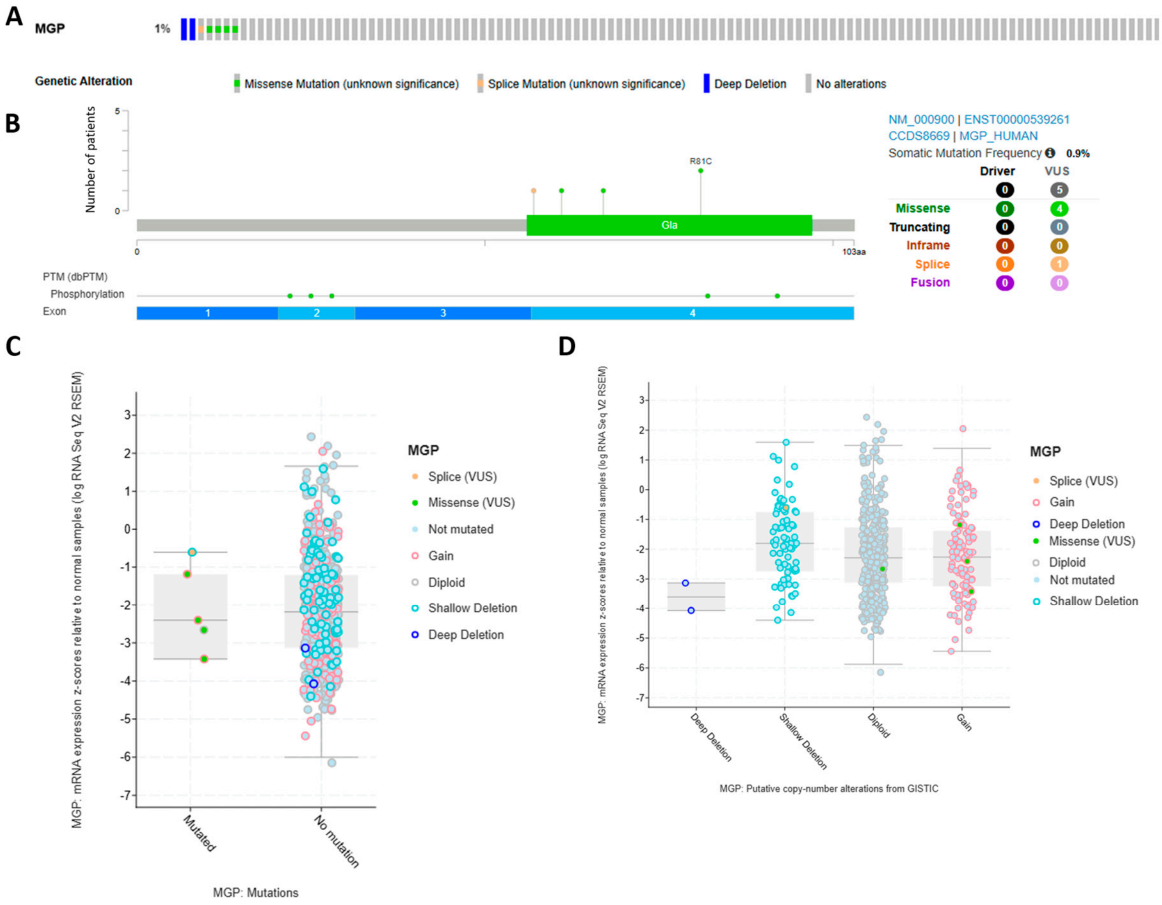

Supplementary Figure S6. Same analysis as Figure S3, but for colorectal cancer samples.

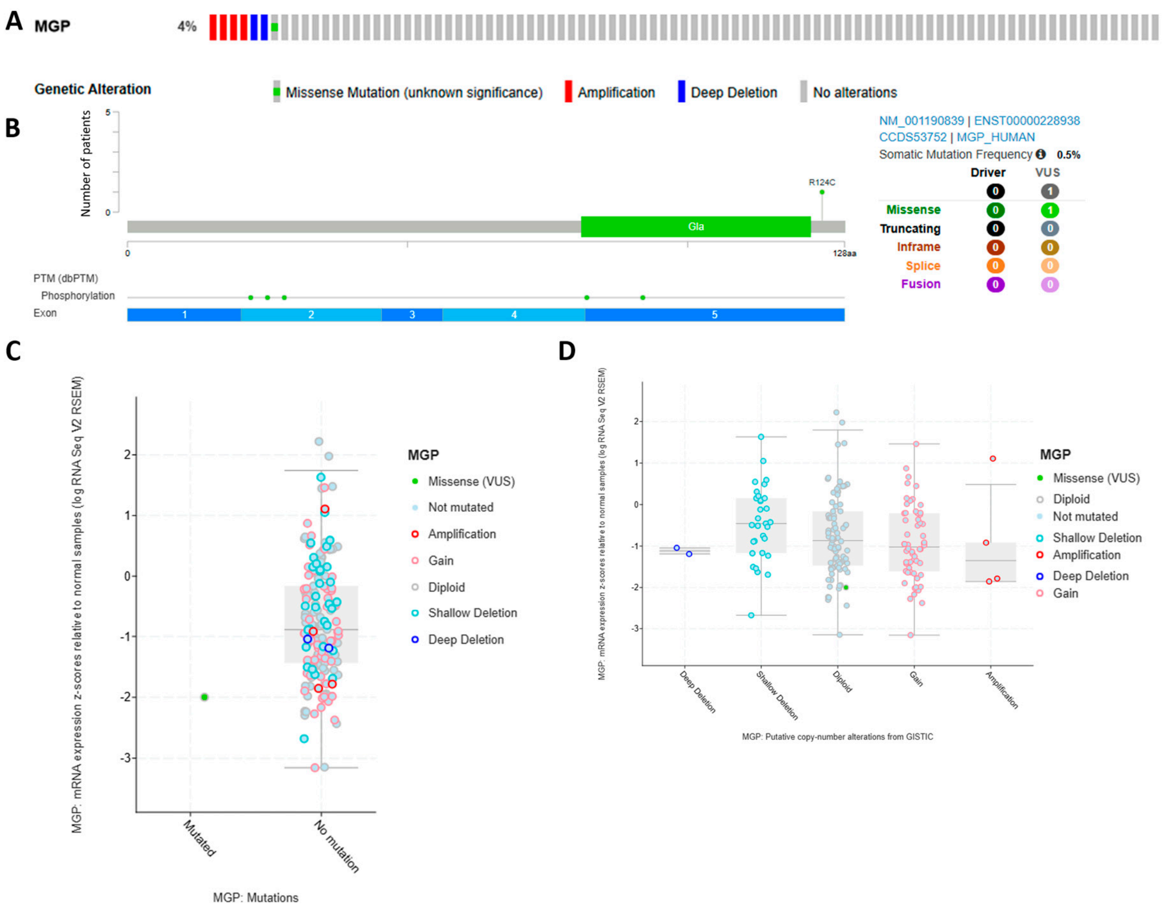

Supplementary Figure S7. Same analysis as Figure S3, but for esophageal cancer samples.

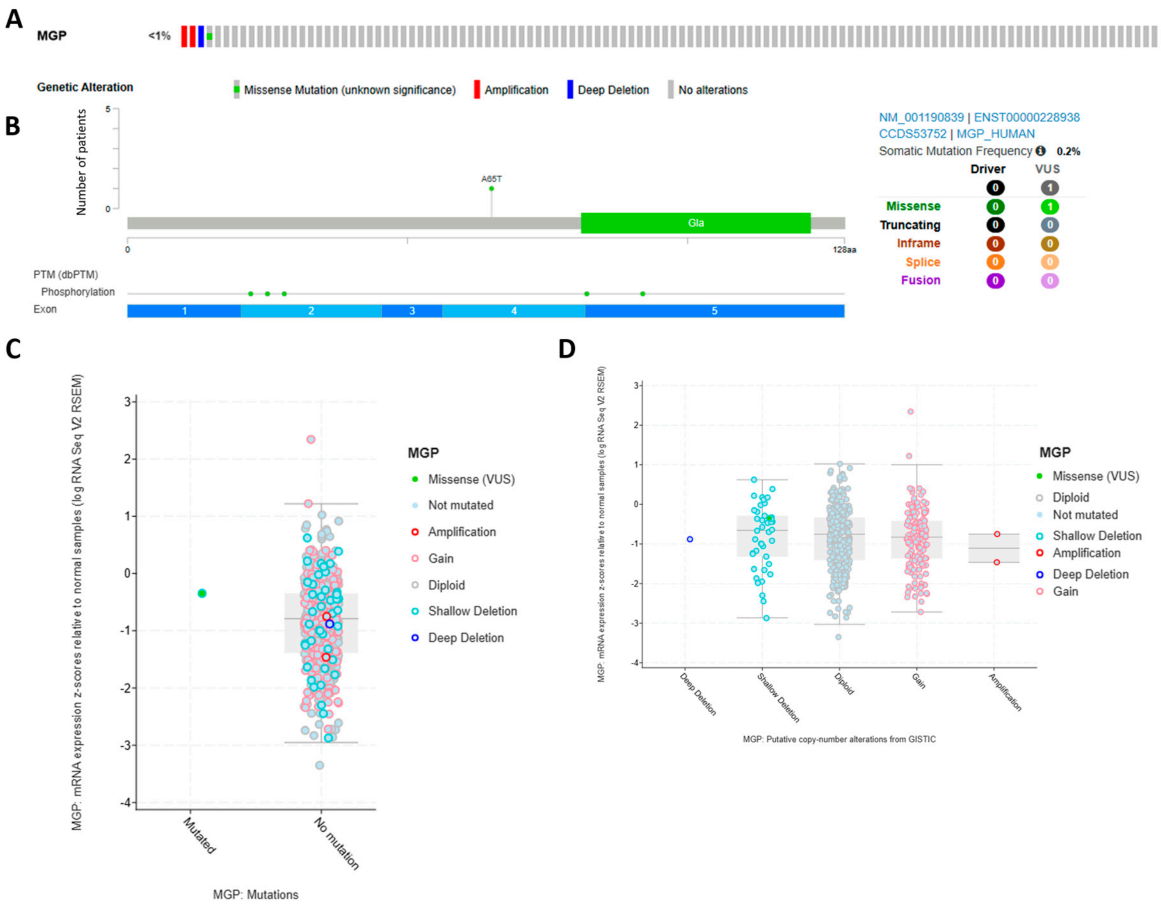

Supplementary Figure S8. Same analysis as Figure S3, but for head and neck cancer samples.

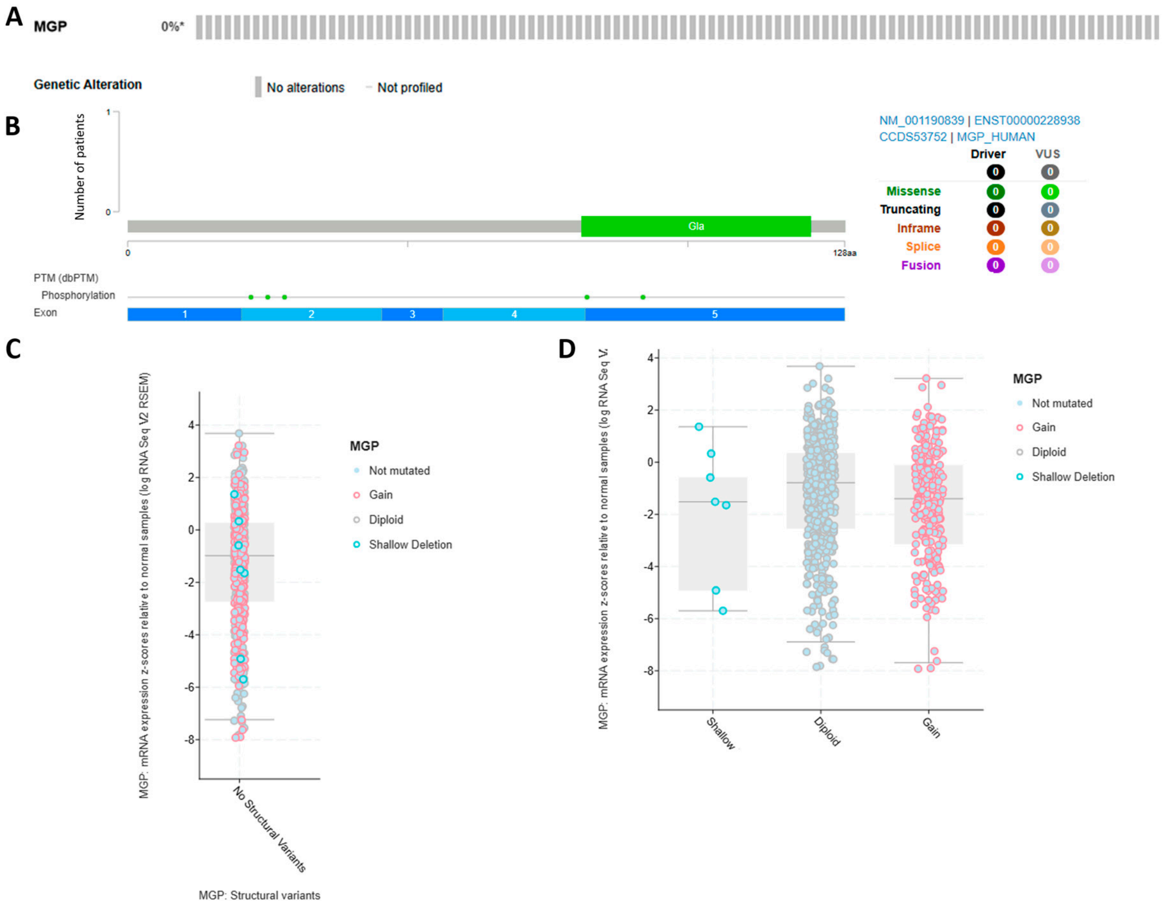

Supplementary Figure S9. Same analysis as Figure S3, but for kidney cancer samples.

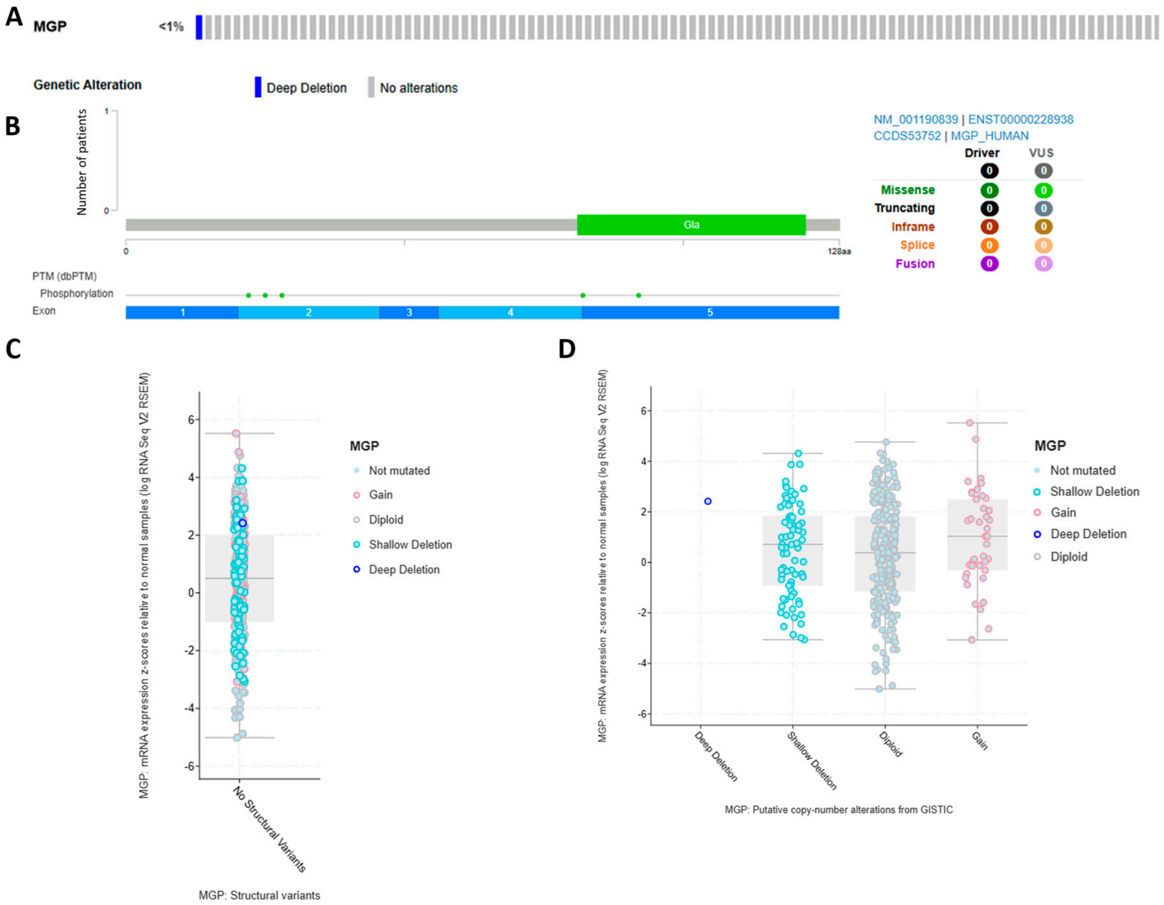

Supplementary Figure S10. Same analysis as Figure S3, but for liver cancer samples.

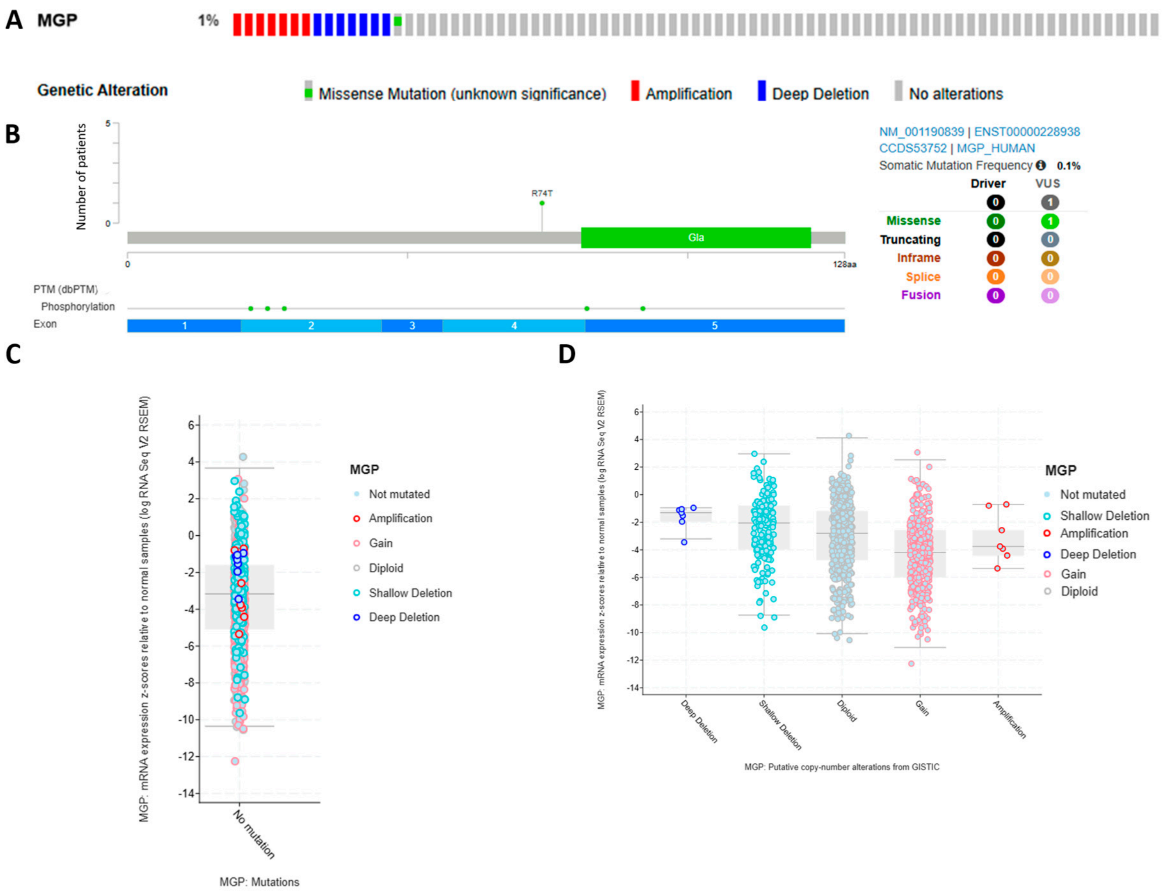

Supplementary Figure S11. Same analysis as Figure S3, but for lung cancer samples.

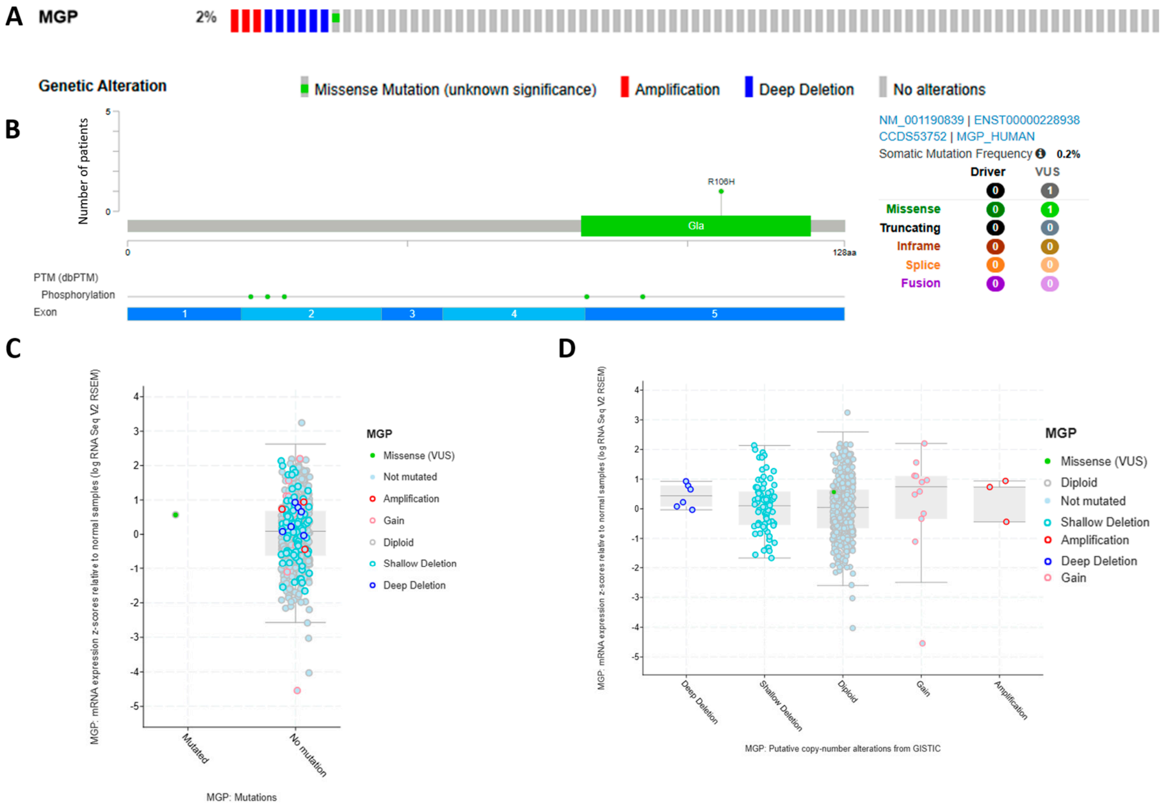

Supplementary Figure S12. Same analysis as Figure S3, but for prostate cancer samples.

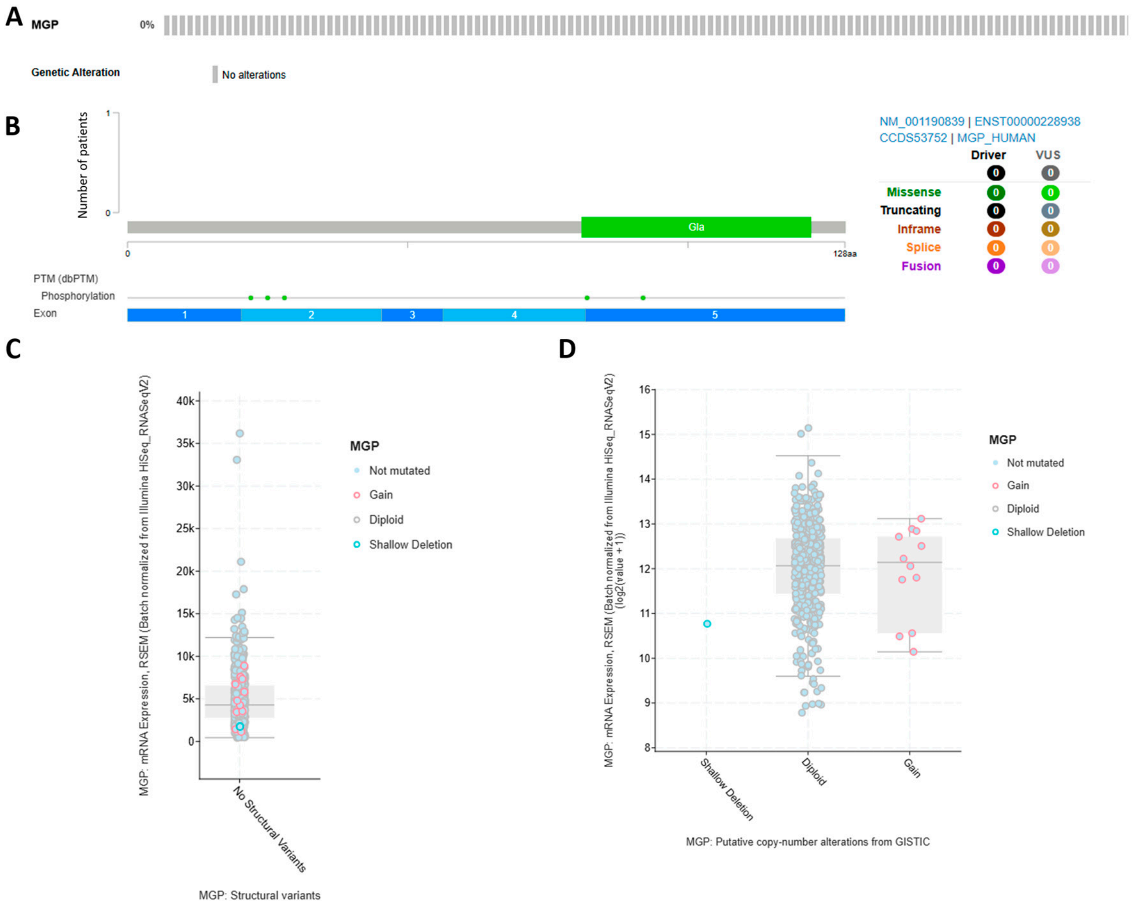

Supplementary Figure S13. Same analysis as Figure S3, but for thyroid cancer samples.
